# Formation of a constructed microbial community in a nutrient-rich environment indicates bacterial interspecific competition

**DOI:** 10.1128/msystems.00006-24

**Published:** 2024-03-12

**Authors:** Jia Wang, Manasa R. Appidi, Leah H. Burdick, Paul E. Abraham, Robert L. Hettich, Dale A. Pelletier, Mitchel J. Doktycz

**Affiliations:** 1 Biosciences Division, Oak Ridge National Laboratory, Oak Ridge, Tennessee, USA; 2 UT-ORNL Graduate School of Genome Science and Technology, University of Tennessee, Knoxville, Tennessee, USA; Purdue University, USA

**Keywords:** microbial community, rhizospheric bacteria, initial inoculum ratio, dynamic flux balance analysis, metabolite exchange, metaproteomics

## Abstract

Understanding the organizational principles of microbial communities is essential for interpreting ecosystem stability. Previous studies have investigated the formation of bacterial communities under nutrient-poor conditions or obligate relationships to observe cooperative interactions among different species. How microorganisms form stabilized communities in nutrient-rich environments, without obligate metabolic interdependency for growth, is still not fully disclosed. In this study, three bacterial strains isolated from the *Populus deltoides* rhizosphere were co-cultured in complex medium, and their growth behavior was tracked. These strains co-exist in mixed culture over serial transfer for multiple growth-dilution cycles. Competition is proposed as an emergent interaction relationship among the three bacteria based on their significantly decreased growth levels. The effects of different initial inoculum ratios, up to three orders of magnitude, on community structure were investigated, and the final compositions of the mixed communities with various starting composition indicate that community structure is not dependent on the initial inoculum ratio. Furthermore, the competitive relationships within the community were not altered by different initial inoculum ratios. The community structure was simulated by generalized Lotka-Volterra and dynamic flux balance analysis to provide mechanistic predictions into emergence of community structure under a nutrient-rich environment. Metaproteomic analyses provide support for the metabolite exchanges predicted by computational modeling and for highly altered physiologies when microbes are grown in co-culture. These findings broaden our understanding of bacterial community dynamics and metabolic diversity in higher-order interactions and could be significant in the management of rhizospheric bacterial communities.

**IMPORTANCE:**

Bacteria naturally co-exist in multispecies consortia, and the ability to engineer such systems can be useful in biotechnology. Despite this, few studies have been performed to understand how bacteria form a stable community and interact with each other under nutrient-rich conditions. In this study, we investigated the effects of initial inoculum ratios on bacterial community structure using a complex medium and found that the initial inoculum ratio has no significant impact on resultant community structure or on interaction patterns between community members. The microbial population profiles were simulated using computational tools in order to understand intermicrobial relationships and to identify potential metabolic exchanges that occur during stabilization of the bacterial community. Studying microbial community assembly processes is essential for understanding fundamental ecological principles in microbial ecosystems and can be critical in predicting microbial community structure and function.

## INTRODUCTION

Multiple species of bacteria exist by living and interacting with each other, and assembling into stable communities with functions that profoundly affect natural environments, agro-ecosystems, and human health (1). For example, numerous bacterial strains form stable communities with plant roots and play pivotal roles in plant growth and health (2, 3). The rhizosphere, the narrow zone of soil around plant roots, is relatively rich in nutrients compared to bulk soil environments and leads to intense microbial colonization and metabolic activities (4, 5). Plant cells supply various metabolites to the rhizosphere, and understanding the ecology in this environment can facilitate the development of management strategies to promote plant growth and to provide sustainable food and renewable energy resources.

Metabolite exchange between species is often considered a major driver in the formation of microbial communities (1). However, experimentally tracking trophic cross-feedings in detail is challenging in rhizospheric microbial communities due to their complex and dynamic nature (2, 6). Synthetic microbial communities with a reduced degree of complexity offer opportunities to disentangle the large web of metabolic interactions that occur during assembly processes (7). In previous studies, simplified communities constructed in nutrient-poor, minimal medium conditions led to emergence of metabolic cross-feedings to maintain genotypic diversity (8). In such a resource-limited environment, species relationships represent the net balance of metabolic interdependencies, leading to robust interspecies mixing (9, 10). In contrast, the incentive for metabolic cross-feeding in nutritionally rich habitats is expected to be lower compared with nutrient-poor environments (11). Meanwhile, ecological competition may still be present in a community since the nutrient-rich niche does not represent an infinite resource stock (12). To date, relatively less attention has been given to how stable bacterial community structure emerges under nutrient-rich conditions, where metabolic interdependencies are theoretically unnecessary for maintenance of biodiversity, in contrast to that observed in nutrient-poor environments (13, 14).

In recent years, computational modeling tools for simulating bacterial growth and cellular physiology have been developed (15). For example, the generalized Lotka-Volterra (gLV) model is a well-known ecological model which predicts interaction types between species and how these interactions affect community diversity (16, 17). Genome-scale metabolic models have been experimentally validated and applied for predicting the presence of metabolic interactions within a microbial community using flux balance analysis (FBA) (18, 19). However, FBA modeling may be insufficient to elucidate temporal metabolic exchanges that occur during community stabilization, since extracellular fluctuations of metabolites cannot be incorporated into steady-state FBA simulations (7, 20). Extension of genome-scale microbial *in silico* models to dynamic flux balance analysis (dFBA) is emerging as a more powerful method to predict the ecological dynamics of microbial communities (21). The dFBA approach can model microbial cell growth and interactions in succession by analyzing temporal changes in the entire set of metabolic fluxes (15, 22). Several dFBA-based simulation software tools have been developed for application to both fundamental and practical problems (23). Among those tools, Computation of Microbial Ecosystems in Time and Space (COMETS) has diverse functional capabilities and can simulate metabolite-mediated emergent interactions during assembly of synthetic microbial communities (24).

In this study, we constructed a synthetic bacterial community as an experimentally tractable model system with the aim of exploring the dynamics and mechanisms of community assembly under nutrient-rich conditions. The selected community consists of three bacterial strains isolated from the *Populus deltoides* rhizosphere and originates from earlier efforts that discovered this stable consortium emerging from a more complex mixture of bacteria (7). Here, these strains were co-cultured using a complex medium and were serially transferred using different initial inoculation ratios to determine their effects on the resulting community structure. R2A is used as a complex medium as it contains diverse substrates for growth, as is typical of the rhizosphere (25), and should reduce the need for obligate metabolic interdependency between community members. The use of different inoculum ratios should provide insight into the role of initial community assembly and diverse limiting nutrients on resultant community structure and on related aspects of priority effects in defining community composition (26–28). To unravel potential metabolic interaction networks, an *in silico* community was constructed and evaluated by gLV and dFBA to predict community dynamics and metabolite exchanges within the tri-culture community. Metaproteomics of the resultant microbial community was carried out to assess the computational modeling predictions by providing metabolic functional information (i.e., proteomes) that could be linked back to the specific taxa.

## RESULTS

### Construction of a three-member community and comparison of the population distributions and growth rates of the component members by plate counting and qPCR

The isolates *Pseudomonas* sp. GM17, *Pantoea* sp. YR343, and *Sphingobium* sp. AP49 were used to construct a synthetic microbial community. In previous work, these isolates were identified as the final surviving species after serially passaging a mixture initially composed of 10 bacteria in a nutrient-rich medium (7). Therefore, we hypothesized that a co-culture consisting of these three bacterial strains can form a stable community in the same medium environment. Meanwhile, competition may be the primary factor influencing the synthetic bacterial community, as all three community members could grow individually in the R2A medium without interdependency (7). An evaluation of pairwise interactions showed that the strain *Pseudomonas* sp. GM17 inhibits the growth of strain *Sphingobium* sp. AP49 (7). For validation, strains GM17, YR343, and AP49 were inoculated into R2A medium with equal proportions and were passaged for five dilution cycles. To assess the resulting populations of the community members, spread plating was used to assess colony-forming units of each species as they could be distinguished based on colony morphology. The succession and relative populations of the three-member bacterial community were also analyzed by quantitative PCR (qPCR). As assessed by either technique, the three strains co-exist throughout the serial dilution process.

**Fig 1:**
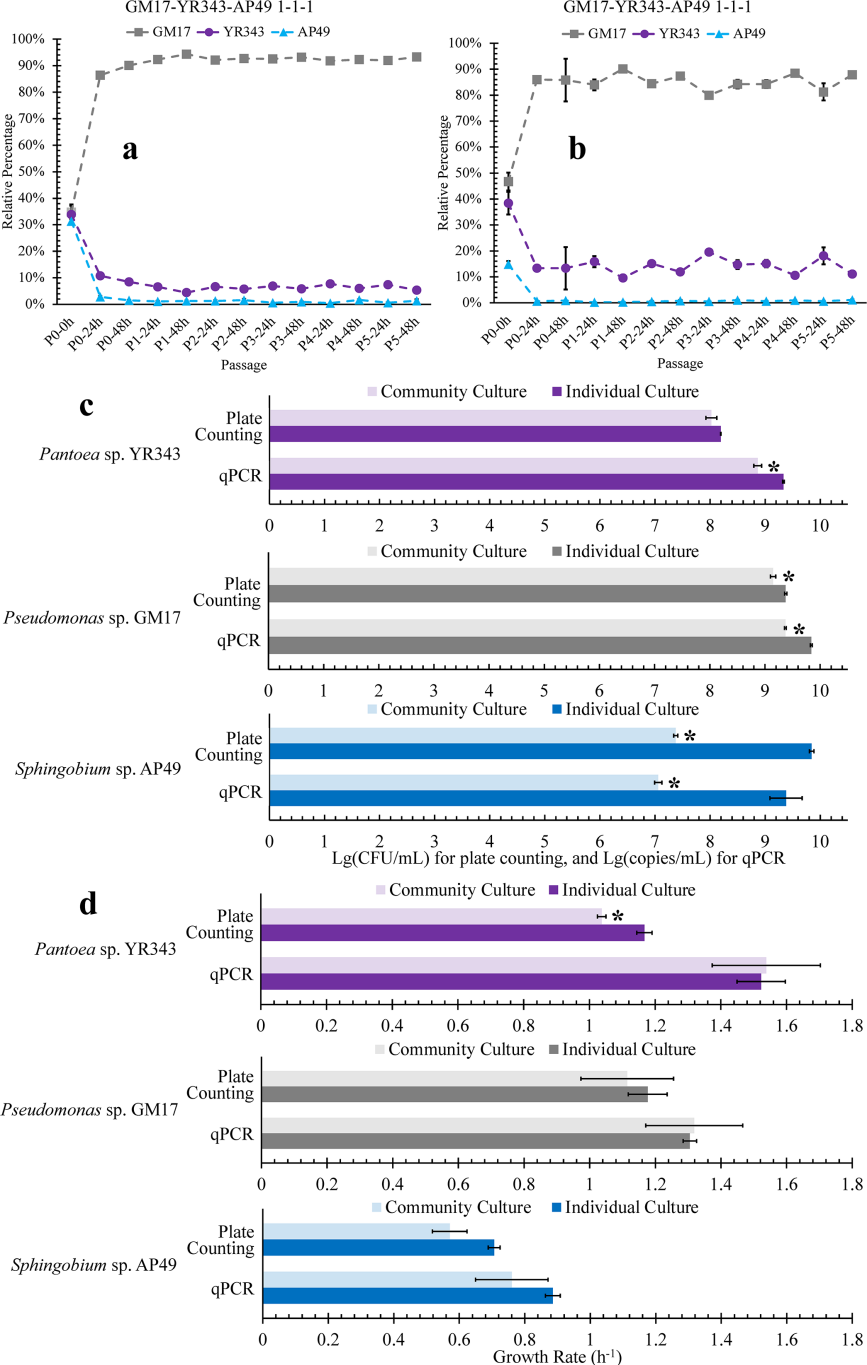
Analysis of the bacterial community structure over assembly process and species growth behavior in passage 0 starting from the inoculum with three species in equal proportion in a nutrient-rich medium environment. The relative abundances of each species in the community are based on (a) plate counting and (b) qPCR method. (c) Comparison of the bacterial cell density values at the end of a growth period between mixed and pure culture modes analyzed by plate counting and qPCR methods. Asterisks show statistically significant decreases in cell abundance compared with monoculture using the same analytical method (*P* < 0.05). (d) Comparison of the maximum growth rate between mixed and pure culture modes determined by plate counting and qPCR methods. Asterisk shows statistically significant decrease in maximum growth rate compared with monoculture using the same analytical method (*P* < 0.05). Each column or data point represents the mean, and error bars are the standard error over three parallel experiments.

The relative percentage for each species in the community was calculated to elucidate temporal variations in community structure at different passages. In the three-member community, *Pseudomonas* sp. GM17 dominates the cell population 24 h after inoculation and keeps its majority presence throughout the following five growth-dilution cycles (Fig. 1a and b). The community structure assessed by both plate counting (Fig. 1a) and the qPCR method (Fig. 1b) shows a similar relative distribution in the percentage of each organism upon community stabilization. When analyzed by qPCR, the relative percentage of strain *Pantoea* sp. YR343 in the stabilized three-member community appears to be larger than that of the result obtained by the plate counting method. Additionally, the qPCR result shows a large deviation from the plate counting method at time 0 h for the community with equal relative ratio of each species.

The growth abundance of each species in the initial passage (P0) was temporally probed to dissect growth behavior during the assembly process. The component organisms’ growth behavior when grown in monoculture, under the same conditions, was used for comparison. Both colony counting and qPCR results identified that the three species in co-culture reached different abundance values that spanned two orders of magnitude, by the end of the growth cycle (Fig. 1c). As the dominant strain in the co-culture, *Pseudomonas* sp. GM17 did not attain the same abundance level compared with its monoculture. When using the colony counting method, *Pantoea* sp. YR343 showed no statistical difference in growth capacity when compared with its growth in monoculture. In general, for the results obtained by qPCR, the genome equivalent of each species in the mixed culture was lower when compared with that of the corresponding monoculture. Among the three species, *Sphingobium* sp. AP49 displays a decrease in abundance of two orders of magnitude when compared with its growth in pure culture when assessed by both the colony counting and qPCR methods. The decrease in the abundance of each species in the constructed community, compared with that of the corresponding monocultures in the same medium environment, indicates competitive relationships within this three-member community. This competitive relationship is further supported by the growth curves of the total biomass in the 3-member community compared to that of the monocultures as assessed by optical density (Fig. S1). It was observed that the overall growth of the 3-member community was lower at the end of initial passage, in contrast to the growth of individual strains *Pseudomonas* sp. GM17 and *Sphingobium* sp. AP49.

Using bacterial abundance measurements obtained by plate counting and qPCR, the maximum growth rate (*µ*_max_) was determined from growth curves for each species in either mixed or pure culture mode (Fig. S2 through S5). The *µ*_max_ of each strain measured by qPCR shows no statistical variation (*P >* 0.05) between growth in mixed and monoculture modes (Fig. 1d). This suggests that competition among these three species does not affect their exponential growth rates in a significant manner even though these bacteria do not attain the same growth abundances as their corresponding monocultures. When assessed by the plate counting method, *Pseudomonas* sp. GM17 and *Sphingobium* sp. AP49 also show no statistical variation in their maximum growth rates when comparing growth in mixed and pure culture modes. In contrast to the qPCR assessment, plate counting indicates that *Pantoea* sp. YR343 has a statistically significantly lower *µ*_max_ in the three-member community when compared with its growth rate in monoculture (Fig. 1d).

### The effect of various initial inoculum ratios on the resulting population structure of the community

**Fig 2:**
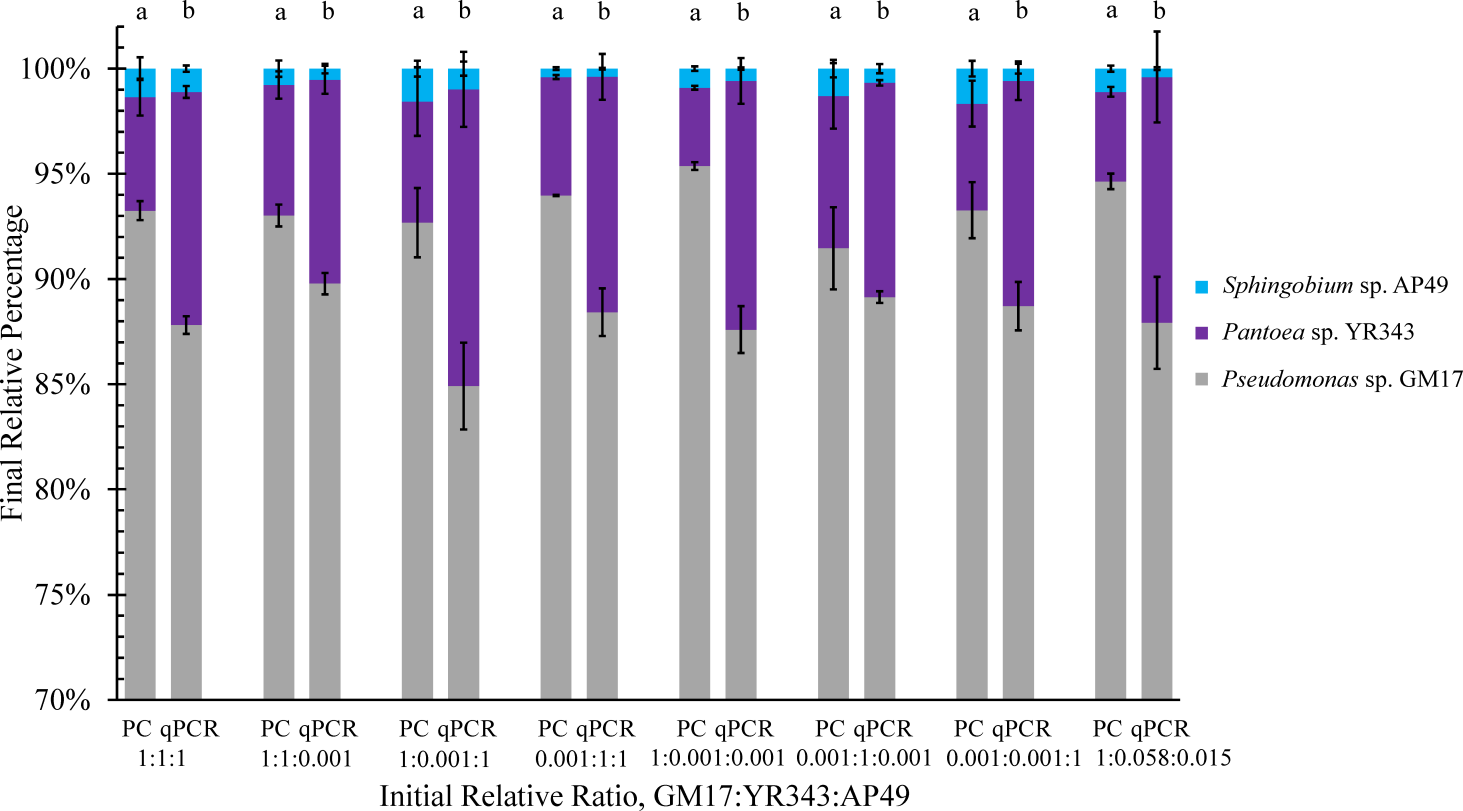
The final relative ratios (at the end of passage 5) of the three strains in the synthetic bacterial communities over a broad range of initial inoculum ratios analyzed by plate counting (PC) and qPCR methods. Each data column represents the mean, and error bars are the standard error over three parallel experiments. Different letters above each column indicate difference of community structure among groups at *P* < 0.05.

To further understand dynamic responses and the potential role of founder effects on the stable 3-member community, a set of communities inoculated at different initial species proportions were constructed using the serial dilution workflow. The absolute abundance of each species in the microbial community was analyzed by both plate counting and qPCR in 24-h intervals throughout the dilution process. Despite 1,000-fold changes in initial inoculum ratios, the bacterial community composition moved toward similar population ratios that were dominated by *Pseudomonas* sp. GM17 (Fig. 2). Although the final population ratio depends on whether plate counting or qPCR is used for the assessment, the final community composition shows no statistical difference across the different initial inoculum ratios when using the same analytical method. These results indicate that the final population ratio does not depend on the relative population level of the initial inoculum. Furthermore, regardless of which strain initially dominates the inoculum, a common population ratio results.

**Fig 3:**
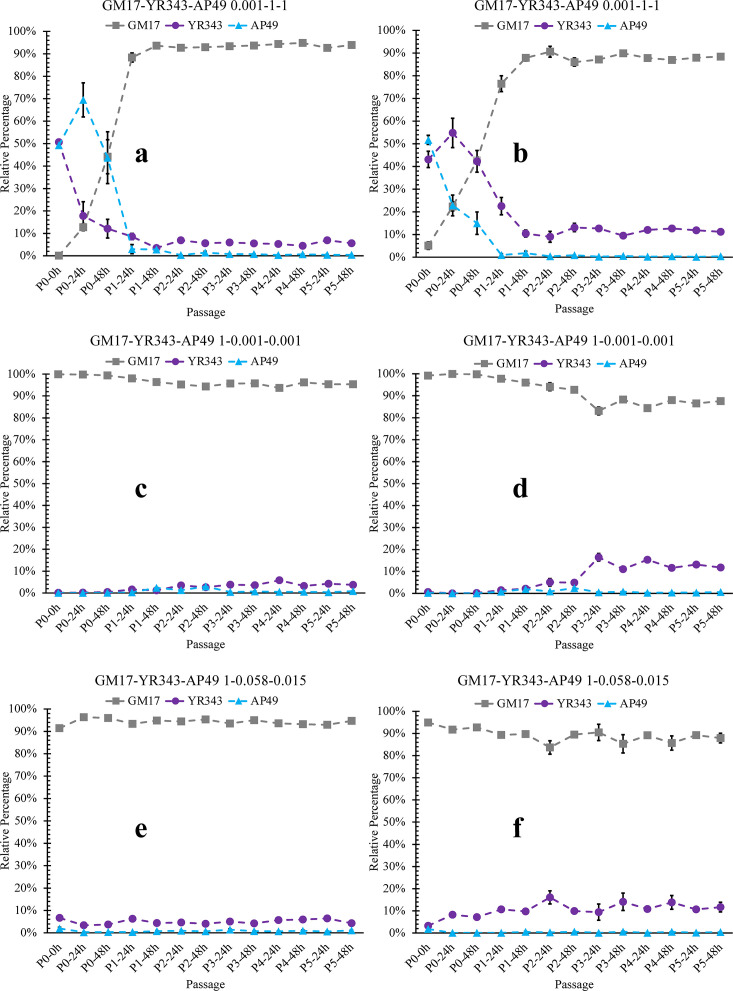
Dynamics of the composition of three-member communities with three representative initial inoculum ratios in a nutrient-rich medium environment. GM17-YR343-AP49 0.001-1-1 analyzed by (a) plate counting and (b) qPCR; GM17-YR343-AP49 1–0.001-0.001 analyzed by (c) plate counting and (d) qPCR; GM17-YR343-AP49 1–0.058-0.015 analyzed by (e) plate counting and (f) qPCR. Each data point represents the mean, and error bars are the standard error over three parallel experiments.

The process of community structure formation over the course of the serial dilution passages for three representative initial inoculum ratios is shown in Fig. 3. With varying species proportions in the inoculum, *Pseudomonas* sp. GM17 was always dominant after five passages. When strain *Pseudomonas* sp. GM17 was diluted 1,000-fold in the initial inoculum, it recovered to a dominant level in two growth-dilution cycles (Fig. 3a and b). When strain *Pseudomonas* sp. GM17 is the major species in the initial inoculum (1.0:0.001:0.001 of GM17:YR343:AP49), it maintained its dominance over the course of the dilution cycles. However, strains YR343 and AP49 maintained presence in the community and increased their relative population levels by more than an order of magnitude by passage No. 2 as determined by both plate counting and qPCR methods (Fig. 3c and d). When starting with an equal initial inoculum ratio, the final composition of the 3-member community was 1.0:0.058:0.015 (GM17:YR343:AP49) as determined by the plate counting method (Fig. 1a). This ratio was applied as an initial inoculum ratio for community assembly, and the community composition showed no variation in subsequent passages (Fig. 3e and f).

Other initial inoculum ratios including 1.0:0.001:1.0, 0.001:1.0:0.001, 1.0:1.0:0.001, and 0.001:0.001:1.0 of GM17:YR343:AP49 were also assessed for their impact on the resulting population structure of the bacterial community (Fig. S6 and S7). In all cases, when *Pseudomonas* sp. GM17 starts at a 1,000-fold dilution level, it recovers its dominance in the community by the end of passage 1. Similarly, regardless of whether *Pantoea* sp. YR343 and *Sphingobium* sp. AP49 start out as dominant or minority components, they return to similar relative ratios after just a couple passages. Both plate counting and qPCR assessment methods show the same population trajectories for the different members of the community. Meanwhile, no statistical differences among all evaluated initial inoculum ratios were found in the final population ratio. Additionally, the absolute abundances of the individual members in the communities were compared with their corresponding abundances when grown in monoculture (Fig. S8). As observed earlier when using a common starting inoculum ratio, decreased cell counts for the individual isolates result when grown in a community setting. This decrease could be attributed to competition among the community members. Negative interactions can also be discerned through a reduction in community productivity when compared to a model that represents the summation of the productivities of the individual species (27, 29). Our results indicate that the productivities of all three-member communities with various starting ratios were significantly lower than the predictions based on the null model (Fig. S9). Meanwhile, the measures of overall biomass for all three-member communities with different initial relative ratios were found to be lower at the end of the assembly process compared to the strain *Sphingobium* sp. AP49, which exhibited the highest growth in monoculture among all three community members in monoculture mode (Fig. S10). These observations are consistent with the competitive interactions detected when comparing the growth level of each specific species in either a community setting or as an individual culture. Collectively, these results indicate that a stable microbiome results when mixing together these three microbes and that the resulting population structure is independent of the relative ratio of the starting inoculum. Furthermore, competition within the members of the community decreases their final population levels.

### Evaluation of interaction type by generalized Lotka-Volterra model

The interspecies interaction coefficients for the gLV models fitted to colony-forming unit (CFU) counting results of community growth under different initial inoculum ratios imply that 85% of the pairwise couplings within the three-member communities are negative interactions. The gLV models derived from qPCR results of growth dynamics estimated that 77% of the pairwise interactions are negative (Fig. S11). These results suggest that the competitive relationship among the bacteria in the three-member community was not significantly changed by different initial inoculum ratios.

### Dynamic flux balance analysis models of community growth and metabolic interactions

**Fig 4:**
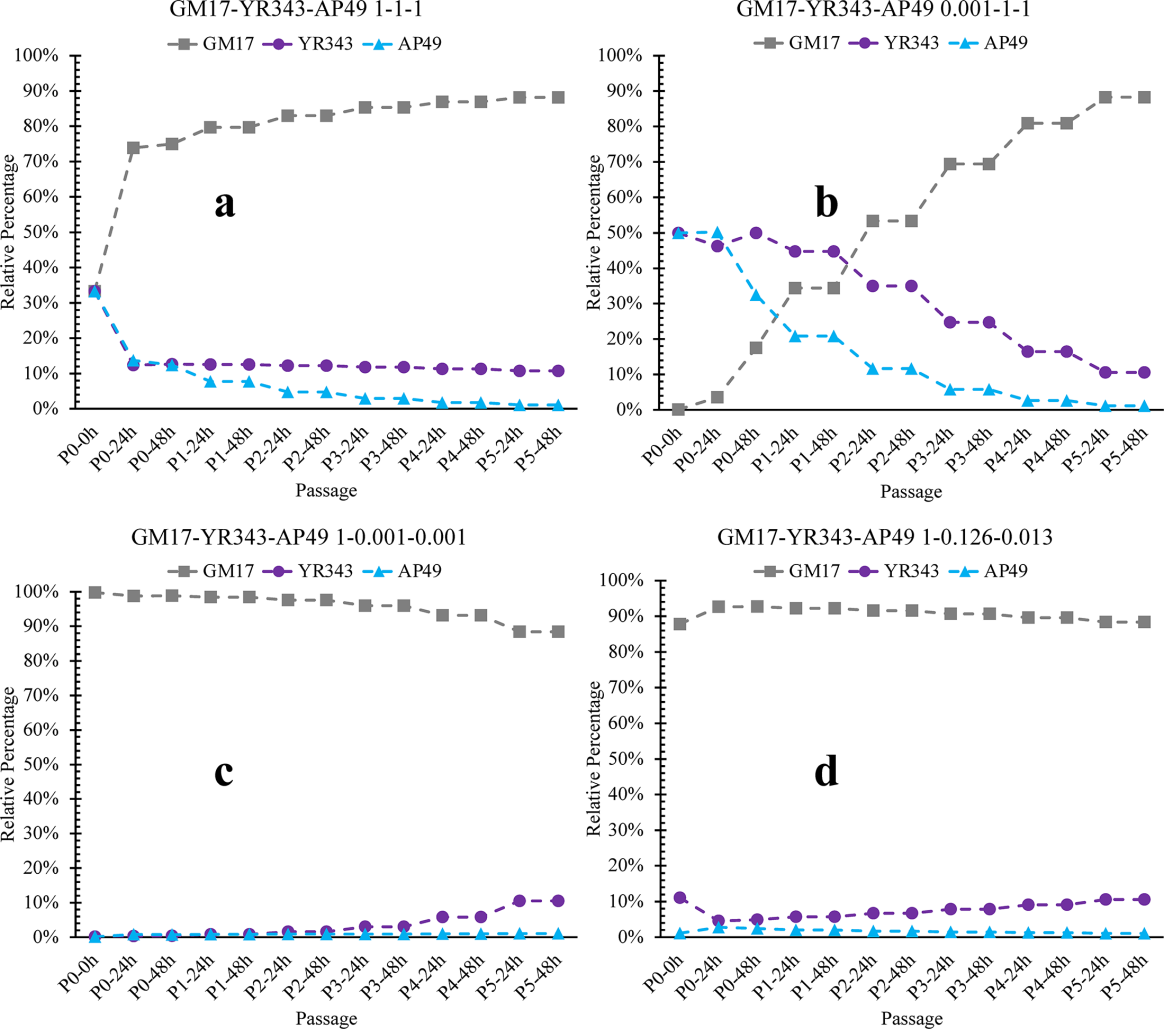
The dFBA model simulations for dynamics of three-member communities with representative initial inoculum ratios (a) 1-1-1, (b) 0.001-1-1, (c) 1–0.001-0.001, and (d) 1–0.126-0.013 of GM17-YR343-AP49.

dFBA accommodates dynamic effects of the extracellular environment on microbial metabolism. Stoichiometric metabolic models of *Pseudomonas* sp. GM17*, Pantoea* sp. YR343, and *Sphingobium* sp. AP49 were used as compartments in the community model. The dFBA predictions for biomass growth of individual cultures generally agreed with the corresponding growth levels obtained from experimental data (Fig. S12). The final composition of the three-member community as measured by the qPCR method (1.0:0.126:0.013 of GM17:YR343:AP49) was used as the reference structure for community dFBA simulations. By changing the maximum uptake rates of limiting nutrients to a lower level, the three-member community dFBA models converged to the same composition by the end of passage 5, even when initial abundances of individual species differed by three orders of magnitude (Fig. 4; Fig. S13). Experimentally, although convergence happens at an earlier passage number, these results suggest that the community dFBA models are able to recapitulate the experimental observations by manipulating the uptake rates of limiting nutrients.

**Fig 5:**
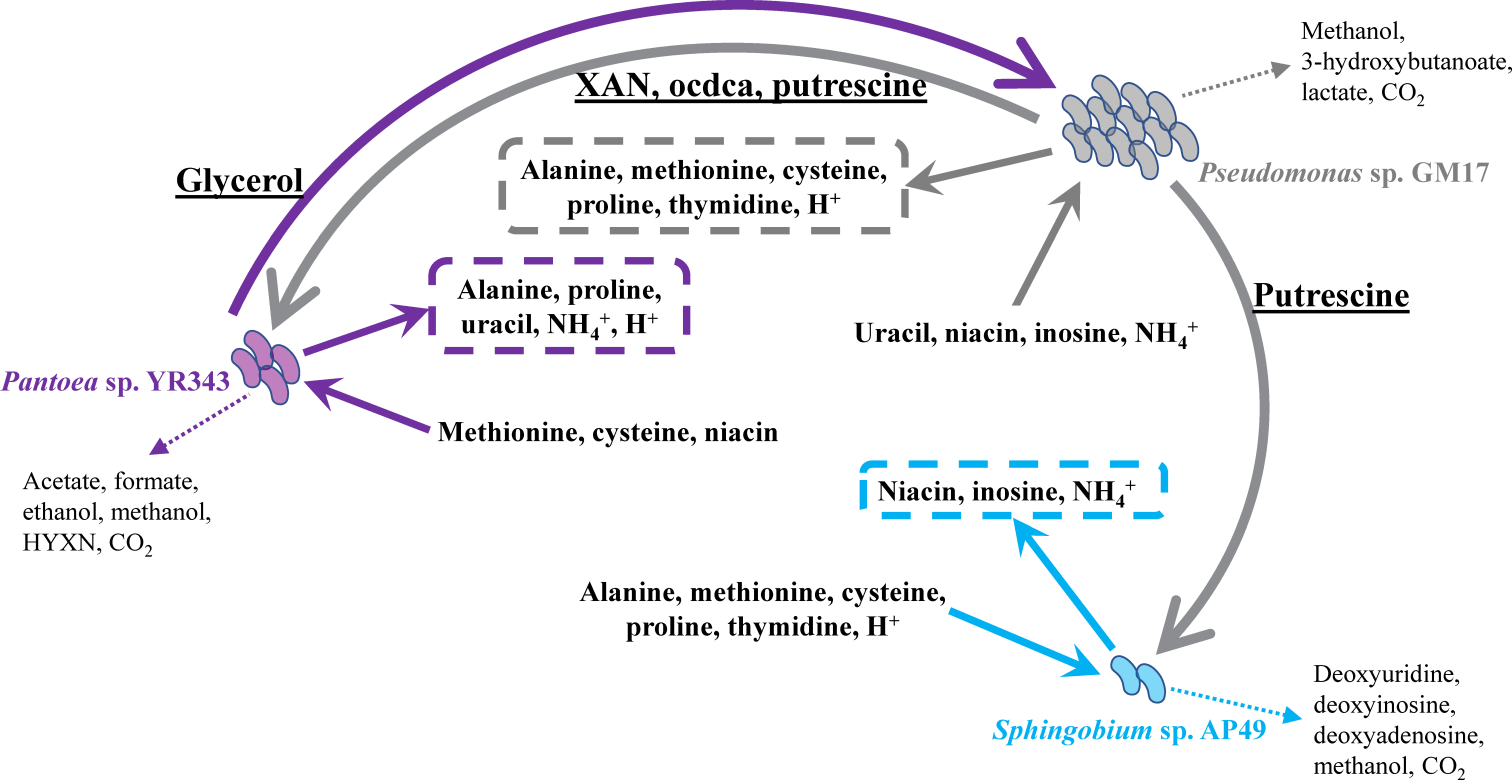
Metabolic interaction network proposed by dFBA simulation in the co-culture with equal initial inoculum ratio. Metabolite abbreviation: HYXN, hypoxanthine.

In order to suggest patterns of metabolic interactions in a nutrient-rich environment, the metabolite exchange fluxes between different species in community dFBA models over six-passage time intervals were captured. The simulations of the three-member communities with equal initial inoculum ratio predict that *Pseudomonas* sp. GM17 secretes xanthine (XAN), octadecanoic acid (ocdca), and putrescine that are consumed by *Pantoea* sp. YR343. In return, strain YR343 is proposed to excrete glycerol, which is consumed by strain GM17. *Sphingobium* sp. AP49 is predicted to uptake putrescine produced by strain GM17 (Fig. 5). The complex medium environment in dFBA models does not contain these metabolites, which are thus considered as potential interspecies metabolic exchanges in the three-member community. When modeled by dFBA in R2A medium as a monoculture, strain YR343 secretes glycerol as a byproduct of its growth, which is consistent with the model prediction in mixed cultures. Simulation of strain GM17 in monoculture also shows that it secretes XAN, ocdca, and putrescine as predicted by community dFBA models. Therefore, the secretions of the metabolites involved in interspecies exchanges by strain YR343 and GM17 are not emergent metabolic fluxes in the three-member community dFBA model under nutrient-rich conditions.

In the community model, *Pseudomonas* sp. GM17 is predicted to secrete four amino acids including alanine, methionine, cysteine and proline, while *Pantoea* sp. YR343 is predicted to excrete alanine and proline into the medium environment. Although *Sphingobium* sp. AP49 is predicted to consume these amino acids, they were not considered as absolute interspecies exchanges since the medium environment also contains these amino acids as nutrients for bacterial growth. Besides amino acids, strain GM17 is predicted to secrete thymidine and H^+^. Strain YR343 is predicted to secrete uracil, NH_4_^+^, and H^+^, and strain AP49 is predicted to secrete niacin, inosine, and NH_4_^+^ to the medium environment. Although different community members are predicted to have uptake fluxes of these compounds and inorganic ions, the medium environment contains all of them as unlimited substrates for bacterial growth. Several other metabolic byproducts were predicted to be secreted into the medium environment but without any uptake by other community members (Fig. 5).

### Examination of metabolic activities of the three-member communities by metaproteomics

**Fig 6:**
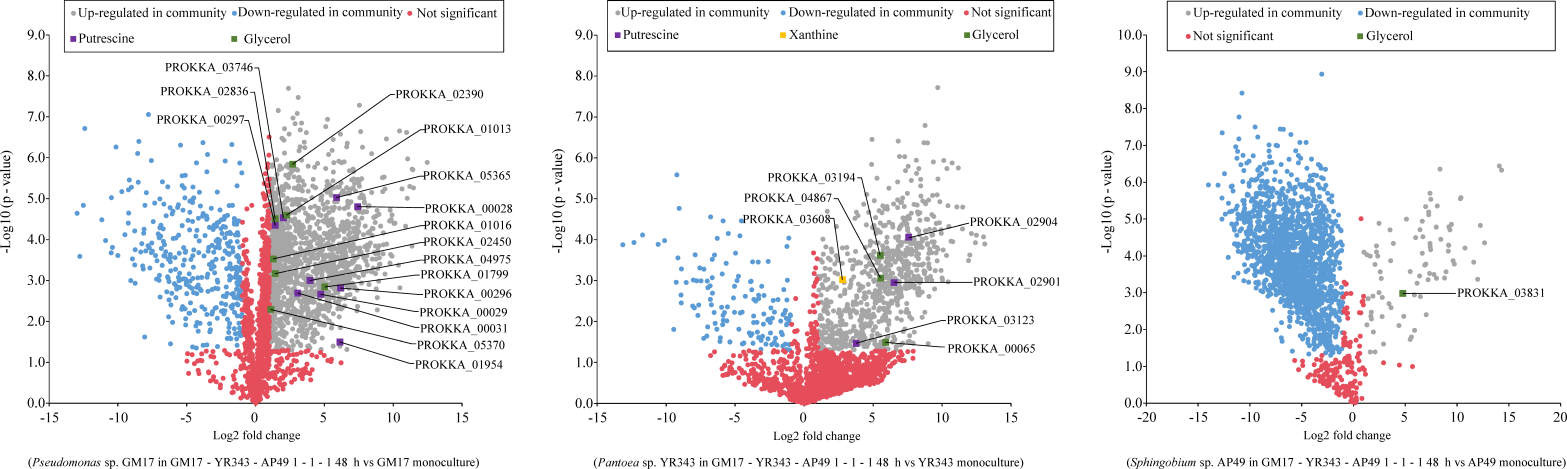
Volcano plots showing the significant differentially abundant proteins for each bacterial species at 48 h in the three-member community with equal initial inoculum ratios (GM17-YR343-AP49 1-1-1) compared to their monocultures at the early stationary phase in R2A medium (a) *Pseudomonas* sp. GM17, (b) *Pantoea* sp. YR343, and (c) *Sphingobium* sp. AP49. Proteins involved in interspecies exchanges as predicted by dFBA analysis are highlighted in the upregulated proteins.

The proteomes of the three bacterial species in the community at 48 h (Table S2) were compared with the proteomic results of the monocultures (Table S3) by performing Student’s *t*-tests to identify the differentially abundant proteins (Fig. 6; Table S4). In the pairwise comparisons between the proteins identified in the monocultures versus community samples, several enzymes involved in biosynthesis of putrescine, glycerol, and other amino acids as predicted by the dFBA model were identified to be significantly more abundant in the community compared to their monocultures. Although the majority of the identified proteins overlapped between the community and monoculture proteomes, a large number of proteins were uniquely identified in the community for strain GM17 and YR343. An opposite trend was observed for strain AP49, where the majority of the proteins were uniquely identified in the monoculture, which could be due to the relatively lower number of proteins detected for *Sphingobium* sp. AP49 than those of *Pseudomonas* sp. GM17 and *Pantoea* sp. YR343 in the community (Fig. S14; Tables S5 and S6).

## DISCUSSION

This study investigated the dynamics of a well-defined bacterial community consisting of three rhizosphere-derived strains, *Pseudomonas* sp. GM17, *Pantoea* sp. YR343, and *Sphingobium* sp. AP49, in a nutrient-rich environment. This system benefits from its relatively simple structure, the availability of genomic information, and previously characterized phenotypic traits (7, 8, 26). Nevertheless, tracking the activities of individual species within even this simple community presents challenges. To understand the growth profiles and bacterial cell concentrations of each species in the community, both CFU-counting and qPCR methods were evaluated. In this work, for strains *Pseudomonas* sp. GM17 and *Sphingobium* sp. AP49, the CFU counting and qPCR techniques provide similar results regarding the absolute abundances and growth rates of the individual organisms when grown in mixed and pure culture. However, measurements for the strain *Pantoea* sp. YR343 show deviation between the plate counting and qPCR methods. This may be due to the accumulation of viable but nonculturable or dead bacterial cells for this strain (30).

Another point of departure is the assessment of community structure at time 0 h in passage 0 when using different initial inoculum ratios. The deviation when using the qPCR method can be attributed to the procedure as the cell concentration of each strain in the inoculum was normalized using the calibration curve between optical density and CFU counts. Detecting small numbers of bacterial cells may also cause potential bias in the presence of a large bacterial cell background (31). As a culture-independent method, qPCR is considered a useful and convenient tool to estimate bacterial cell amount (32, 33). PCR bias has no impact on the qPCR method since a calibration curve is generated using the target strain with known cell concentration (34). Provided suitable primers are available, qPCR is better suited for complex mixtures of microbes as distinguishing more than a few different colonies by morphology can be challenging. However, the ability to distinguish live from dead cells can be a shortcoming of PCR-based characterization. Regardless, both the plate counting and qPCR techniques indicate that a consistent community structure results upon combining and growing these microbes in rich media.

A surprising result is the observation that a similar community population structure emerges despite broad ranges of initial inoculum ratios. Apparently, 1,000-fold changes in starting inoculum amounts do not impact the final community population structure. The initial inoculation ratio has been shown to have a nontrivial influence on the structures of simple co-culture communities with two different bacterial members, and the dominant species in a community can regulate the gene expression of minor species even at the beginning phase of growth (26, 35, 36). Comparisons of the growth curves for the individual species when grown by themselves and in a community indicate that their maximum growth rates are approximately the same, but their biomass yield when in a mixed community is reduced. The lower biomass level of each species in the community compared with their corresponding monocultures suggests that mutual benefits associated with metabolite exchanges between species are unlikely. The serial transfer regime employed allows for depletion of a limiting substrate during the growth period and for the bacteria to co-exist as a community with negative interactions (37–39).

The computational model-based analysis provides systematic interpretation which can promote mechanistic understanding of potential cross-feeding interactions in microbial communities (40). As an ecological model, the gLV approach can be used to reflect either positive or negative effects of species on one another by fitting temporal dynamics of species abundances (41). In this study, gLV modeling results support the competitive relationships implied by the experimental results that determined a decreased biomass yield of each species in community cultures with different initial inoculum ratios. The emergent composition of microbial communities is thought to be largely shaped by exchanges of metabolites during community assembly (11, 42). Generalized Lotka-Volterra model can only describe pairwise interactions without any integration of metabolite exchanges (17). The use of dFBA models enables the tracking of temporal fluxes of secretion and uptake of a complete set of metabolites which are not computationally tractable by gLV or steady-state FBA models (43). Although dFBA models continue to advance, their accuracy for emergent community composition remains as a challenge (23, 44). The experimentally obtained community dynamics is highly recommended for use as a priori knowledge to infer metabolic interactions in the modeling framework (43).

Metaproteomics analysis allowed for detection of hundreds of proteins in both isolates and community samples, where the majority of proteins overlapped between the two conditions for *Pantoea* sp. YR343 and *Pseudomonas* sp. GM17. However, there were a significant number of proteins that were uniquely identified and differentially expressed in the community compared to monocultures, indicating significant metabolic changes when microbes are grown in co-culture. Proteins involved in the biosynthesis of specific metabolites, putrescine and glycerol, that are predicted to be exchanged by dFBA analysis are among these differentially expressed proteins. Indeed, several of these identified proteins were observed to be significantly more abundant in the three-member community compared to their monocultures for *Pantoea* sp. YR343 and *Pseudomonas* sp. GM17. The high representation of *Pantoea* sp. YR343 and *Pseudomonas* sp. GM17 in the metaproteomics data allowed for detection of several significantly differentially abundant proteins in the community, as predicted by the dFBA model analysis. In addition to these metabolites, several proteins involved in the arginine and proline metabolism, purine metabolism, fatty acid biosynthesis, fatty acid elongation, fatty acid degradation, and glycerolipid metabolism were identified to be significantly differentially abundant in the community samples (Tables S5 and S6). The metaproteomic analysis is consistent with the interactions predicted by dFBA, as proteins involved in the biosynthesis and uptake of the exchanged metabolites can be identified in both GM17 and YR343.

The simple microcosm setup described in this study allows connection between community assembly outcomes and genome-scale metabolic models. Furthermore, species-level dynamics in the synthetic bacterial community can be observed, offering the opportunity to reveal the interaction patterns and the effects of growth rate of the individual species in co-culture. In this multispecies model system, no significant trade-off between a species’ maximum growth rate and its ultimate existence was observed under nutrient-rich conditions. Furthermore, a wide range of initial inoculum ratios of the component members did not significantly change the stabilized community structure or the competitive interaction patterns observed in the community. The dFBA modeling of community successional dynamics was consistent with experimental profiles, providing putative mechanistic insights related to metabolic interactions in the co-cultivation of strain *Pseudomonas* sp. GM17, *Pantoea* sp. YR343, and *Sphingobium* sp. AP49 in a nutrient-rich environment. Metaproteomics results support the predicted metabolic interactions and, importantly, define the extensive proteome remodeling that occurs when an organism grows in a multispecies community under nutrient-rich conditions. Simple constructed communities such as the one described here, along with effective analytical and modeling tools, can improve our basic understanding of synthetic ecology and provide a starting point for designing and controlling microbial communities for desired biological functions.

## MATERIALS AND METHODS

### Community construction

The bacterial strains *Pseudomonas* sp. GM17, *Pantoea* sp. YR343, and *Sphingobium* sp. AP49 were previously isolated from the rhizosphere of *Populus deltoides*, and draft genome sequences were reported (7, 45). Bacterial monocultures were inoculated from R2A agar plates and grown in 10 mL of R2A medium in test tubes overnight at 30°C with shaking at 200 rpm for preparing seed cultures. The R2A complex medium was prepared as described in reference (46). For community construction, each species was normalized to create a bacterial population of 10^7^ CFU/mL, using a calibration curve between OD_600_ and CFU/mL established for each strain (Fig. S15). An initial inoculum was prepared by mixing 300 µL of overnight cultures of the three strains at equal ratios (100 µL of each strain) in 9.7 mL of R2A medium and cultivated at 30°C with shaking at 200 rpm. Every 48 h, 10% (vol/vol) of the culture was transferred to a new test tube carrying 9-mL fresh R2A medium. Triplicate test tubes were subjected to serial growth experiments. At the 24- and 48-h time points of each growth cycle, both spread plate-based CFU counting on R2A and qPCR were used to assay the population of each species. The relative percentage of each species was computed as the read counts or copies for each strain divided by the total number of reads or copies at each time point.

### Genomic DNA sequencing

To obtain improved quality genome sequences, from *Pseudomonas* sp. GM17, *Pantoea* sp. YR343, and *Sphingobium* sp. AP49, gDNA PacBio SMRT long-read sequencing was performed at SNPsaurus (Eugene, OR). Genomic DNA was extracted from 4 mL of overnight cultures grown in R2A medium. DNA extractions were performed using Qiagen DNeasy kit (Qiagen, Germantown, MD) following the manufacturer’s instructions. Flye v.2.8 was used to assemble and polish the sequenced genomes (47). The assembly quality was assessed using BUSCO v.4 (48). An average genome coverage of approximately 270-fold was obtained for each genome. The assemblies were annotated using Prokka with default parameters (49).

### Species-specific quantitative PCR

Bacterial cells were collected by centrifugation at 12,000 rpm for 15 min. Total DNA was extracted using a Qiagen DNA Isolation kit (Qiagen) as per the manufacturer’s instructions. The extracted DNA was used as a template for the qPCR process. The concentration of DNA templates was measured using a Nanodrop system (Thermo Scientific, Wilmington, DE). Each qPCR mixture (20 µL) was composed of 10 µL of SYBR Green PCR Master Mix (Bio-Rad, Hercules, CA), 4 µL of primer mixture (5 mM for forward and reverse primers), 5 µL of H_2_O, and 1 µL of DNA template. The qPCR conditions were as follows: initial denaturation at 95°C for 5 min, followed by 45 cycles of 95°C for 30 s and 60° for 45 s. Quantitative PCR amplification was performed using a CFX96 Real-Time System (Bio-Rad) and monitored with the Bio-Rad CFX manager v.3.1 software. The primers used for qPCR analysis in this work are shown in Table S1. The standard curves were generated using triplicate 10-fold dilutions of the DNA extracted from each species. Species abundance was expressed as the number of genome equivalents calculated from qPCR amplification of genomic DNA.

### Microbial community culturing at different initial inoculum ratios

The tri-culture community system was constructed under eight different initial inoculum ratios. In addition to an initially equal proportion, other initial inoculum ratios include 1.0:1.0:0.001, 1.0:0.001:1.0, 0.001:1.0:1.0, 1.0:0.001:0.001, 0.001:1.0:0.001, 0.001:0.001:1.0, and 1:0.058:0.015 of *Pseudomonas* sp. GM17:*Pantoea* sp. YR343:*Sphingobium* sp. AP49. The initial ratio of 1.0:0.058:0.015 is the observed, stabilized community ratio when starting with equal amounts of the three species in the initial inoculation. To prepare an initial inoculum, an overnight culture of each bacterium was normalized to 10^7^ CFU/mL by calibration curve between OD_600_ and CFU/mL, and then one or two of the corresponding strains were diluted 1,000-fold to 10^4^ CFU/mL. One hundred microliters of each component was combined with 9.7-mL R2A liquid medium to attain a final volume of 10 mL. Triplicate samples were subjected to serial growth experiments. The population densities for each of the three strains were tracked over six growth-dilution cycles, and the community structures were determined by both plate counting and qPCR methods.

### Determination of maximum specific growth rate and comparison of growth abundance of each species in mixed and monoculture

The maximum growth rate of each species in the mixed culture was determined from growth curves derived from species-specific CFU counting and qPCR protocols. For determining the maximum growth rate of each species in monoculture, 0.1 mL of each strain was normalized to 10^7^ CFU/mL and inoculated into 9.9 mL R2A medium. The number of cells was monitored by both CFU counting and qPCR methods to generate the growth curve. The maximum growth rate of each bacterial strain was calculated as the slope of the plot between ln(*X*/*X*_0_) and the time during the exponential growth phase, where *X* is the cell mass at *t* and *X*_0_ = initial cell mass. The increase in population size between *t* = 0 and 48 h, that is, *N_t_*_=48_/*N_t_*_=0_, was calculated using both CFU counting and qPCR results for cell abundance.

### Determination of interaction type by the gLV model

The gLV model was simulated using a time-series data set of microbial abundance of community members during serial dilution processes by Metagenomic Microbial Interaction Simulator software (50). The mean abundance of species at each time point over three parallel experiments was applied as input for gLV modeling, and the interaction coefficient calculated by gLV model was used to infer a positive or negative interaction type.

### dFBA using COMETS toolbox

The genomes of the three strains were used to reconstruct sequence-specific metabolic networks using the Department of Energy Systems Biology Knowledgebase (KBase) “Annotate Genome/Assembly with RASTtk” and “Build Metabolic Model” applications (51–53). The metabolic models were all gap-filled in the R2A medium environment. The KBase narrative containing the genomes and metabolic models can be found at https://kbase.us/n/117918/17/, https://kbase.us/n/117919/16/, and https://kbase.us/n/117923/15/.

The different species were incorporated into COMETS in Python for community dFBA simulations by importing the corresponding metabolic models from KBase pipelines. The dFBA simulations for the tri-species communities with different initial inoculum ratios were all performed using the parsimonious algorithm (24, 54). Glucose and amino acids were considered as the limiting carbon sources in the R2A complex medium environment. The initial molar amount of glucose was calculated based on the glucose content in the R2A medium composition. The amino acids were considered as equally distributed in peptone, casamino acids, and yeast extract by weight, and they were exhibited as molar weight for their initial composition in the R2A medium. All other inorganic ions, gases, and growth factors were set as unlimited throughout all the simulations. The serial transfer of the community was simulated by COMETS using batch dilution function, and the dilution factor was set as 0.1, which is the same as the serial dilution experiment for the community assembly process. The initial biomass of each strain in the *in silico* community was defined to match the initial inoculum amount and ratios used in community assembly experiments.

The dFBA framework for modeling the three-member bacterial communities was calibrated to experimental data for the final community composition obtained by the qPCR method. To fit the simulated, stabilized community structure to the experimental results, the uptake rates of the carbon sources for each species in the simulations of communities were adjusted. The uptake rates of glucose and all amino acids for a community member were changed simultaneously to the same value. The limiting carbon source (glucose and amino acids) uptake rates of all three species in the community were first set at 10 mmol/gDW/h. The limiting carbon source uptake rates of strain *Pantoea* sp. YR343 and *Sphingobium* sp. AP49 decreased simultaneously from 10 to 1 mmol/gDW/h with a decrement of 1 mmol/gDW/h until reaching 1 mmol/gDW/h, and then decreased with a decrement of 0.1 mmol/gDW/h. The uptake and secretion values for each community model are listed in Tables S7 through S9. If the final biomass percentage of a species in the simulation was lower than 95% of its final biomass percentage after six passages in the experiment, its limiting carbon source uptake rates were returned to the last value and decreased again with a decrement of 0.01 mmol/gDW/h or 0.001 mmol/gDW/h. When the final biomass percentage of every species in the community model was in the range of 95%–105% of the biomass percentage of corresponding species in the stabilized community after six passages in the experiment, the simulation was then considered as successfully fitting to the experimental data. The default *V*_max_ of each strain in the community model for all other unlimited nutrients was set to 10 mmol/gDW/h. The initial cell dry mass was 3.9 × 10^−7^ g, and if a species was 1,000-fold diluted at the starting point, its initial cell dry mass was set as 3.9 × 10^−10^ g. The fluxes of metabolites involved in interspecies exchanges were extracted using the method in reference (24). The growth dynamics of the monoculture of each species was also simulated using the dFBA model, and the metabolite exchange fluxes between the bacterial cell and the medium environment were extracted for further comparison with those exchange fluxes in the community models.

### Metaproteomic analysis

#### Microbial community culturing for proteomic analysis

A three-species community system was constructed as described above under microbial community culturing at an initial inoculum ratio of 1:1:1 of *Pantoea* sp. YR343:*Pseudomonas* sp. GM17:*Sphingobium* sp. AP49. The three bacterial strains were also grown in isolation. All cultures were grown in triplicate in R2A media at 30°C with shaking at 200 rpm. Individual cultures were collected at an early stationary phase, and community samples were collected at 48 h. Cell pellets were stored at −80°C.

#### Cellular protein extraction

All cell pellets were solubilized in 300 µL of lysis buffer [4% sodium dodecyl sulfate (SDS) in 100-mM Tris, pH 8.0], followed by sonication (30% amplitude, 10-s pulse with 10-s rest, 2-min total pulse time using a Branson digital sonifier; Branson Ultrasonics Corporation, CT) and incubation at 90°C for 10 min. The cell lysates were centrifuged at 21,000 × *g* for 10 min at room temperature to remove any cell debris, and proteins were quantified with a Nanodrop One spectrophotometer (Thermo Scientific). Samples were reduced and alkylated, followed by protein extraction by protein aggregation capture method and enzymatic tryptic digestion as previously described (55). The samples were adjusted to 0.1% formic acid (FA) (Fisher Chemical, Fair Lawn, NJ), vortexed, and allowed to sit for 10 min. The samples were placed on a magnetic rack, and supernatants containing the peptides were added to prewet Vivaspin 10-kDa molecular weight cut off (MWCO) filters (Sartorius, Bohemia, NY) and centrifuged at 12,000 × *g* for 15 min at room temperature. Tryptic peptide flow throughs were collected, and peptide concentrations were measured using the Nanodrop One spectrophotometer (Thermo Scientific) and transferred to the autosampler vials for liquid chromatography-tandem mass spectrometry (LC-MS/MS) measurements.

#### Protein identification and quantification

All peptide samples were analyzed as previously described using two-dimensional LC-MS/MS with a few modifications in separation (55). Community sample peptide mixtures were separated and analyzed across three successive strong cation exchange (SCX) fractions of increasing concentrations of ammonium acetate (35mM, 50mM, and 1M). The bacterial isolate peptide mixtures were separated using the same separation regime, albeit with only one SCX fraction (1M). All MS raw data files were analyzed using the Proteome Discoverer software v.2.5 (Thermo Fisher Scientific, Cleveland, OH), and statistical analysis was performed as previously described (55). Pairwise comparisons were performed for each bacterial strain to identify the differences between the community and isolate proteomes.

## Data Availability

All proteomics spectral data in this study were deposited at the ProteomeXchange Consortium via the MassIVE repository (https://massive.ucsd.edu/). The ProteomeXchange project identifier is PXD042060, and the data can be reviewed under the user name “MSV000091881_reviewer” and password “PMI_3-member.” The genome sequences and assemblies have been deposited at National Center for Biotechnology Information (NCBI) and can be found at NCBI under BioProject accession number PRJNA83069 (*Pseudomonas* sp. GM17), PRJNA83107 (*Pantoea* sp. YR343), and PRJNA83037 (*Sphingobium* sp. AP49). The Python v.3.8.8 code for dFBA modeling is available in the GitHub repository: https://github.com/Wangjia6555/3-Member_Community_in_R2A.

## SUPPLEMENTAL MATERIAL

Supplemental figures and table msystems.00006-24-s0001.pdf Figures S1-S15 and Table S1.

Table S2 msystems.00006-24-s0002.xlsx Metaproteomics.

Table S3 msystems.00006-24-s0003.xlsx Proteomics.

Table S4 msystems.00006-24-s0004.xlsx *t*-Test results.

Table S5 msystems.00006-24-s0005.xlsx Proteomics summary.

Table S6 msystems.00006-24-s0006.xlsx Metaproteomics summary.

Table S7 msystems.00006-24-s0007.xlsx Uptake rates for *Pseudomonas*.

Table S8 msystems.00006-24-s0008.xlsx Uptake rates for *Pantoea*.

Table S9 msystems.00006-24-s0009.xlsx Uptake rates for *Sphingobium*.

ASM does not own the copyrights to Supplemental Material that may be linked to, or accessed through, an article. The authors have granted ASM a non-exclusive, world-wide license to publish the Supplemental Material files. Please contact the corresponding author directly for reuse.
