# Supplementary material for: Formation of a constructed microbial community in a nutrient-rich environment indicates bacterial interspecific competition
Source: mSystems. 2024 Mar 12;9(4):e00006-24. doi: 10.1128/msystems.00006-24 (PMC11019790; doi:10.1128/msystems.00006-24)
Supplement: Supplemental figures and table — Figures S1-S15 and Table S1. [file msystems.00006-24-s0001.pdf]

Supplementary Material

Formation and interaction network of a constructed microbial community in a nutrient-rich environment

Jia Wang[a,c], Leah H. Burdick[a], Manasa R. Appidi[a,b], Paul E. Abraham[a], Robert L. Hettich[a], Dale A. Pelletier[a,*], Mitchel J. Doktycz[a,*]

[a]Biosciences Division, Oak Ridge National Laboratory, Oak Ridge, TN, USA

[b]Graduate School of Genome Science and Technology, University of Tennessee, Knoxville, TN, USA

[c]Current address: Department of Food Science, University of Tennessee, Knoxville, TN, USA

* Corresponding authors

E-mail addresses: pelletierda@ornl.gov (D. A. Pelletier) and doktyczmj@ornl.gov (M. J. Doktycz)

Table S1. List of qPCR primers used in this study

Microbial target	Primer sequence (5'-3')	Length (nt)	T_m (°C)
Pseudomonas sp. GM17 16S rRNA (1)	F: TGTCACTATTATCAGCCATTGTAGA	25	59.7
	R: AACAGTGGATGAGGTCTAATAACAA	25	59.7
Pantoea sp. YR343 16S rRNA	F: GCGTAACTTCATTGAGTAGCTTTAC	25	61.3
	R: GTGGAATGCTTAACTTTGTTACCTA	25	59.7
Sphingobium sp. AP49 16S rRNA	F: GACAGGAGCAACGCCTATAA	20	60.4
	R: CAATGGACGATGCTGTTGAAG	21	60.6

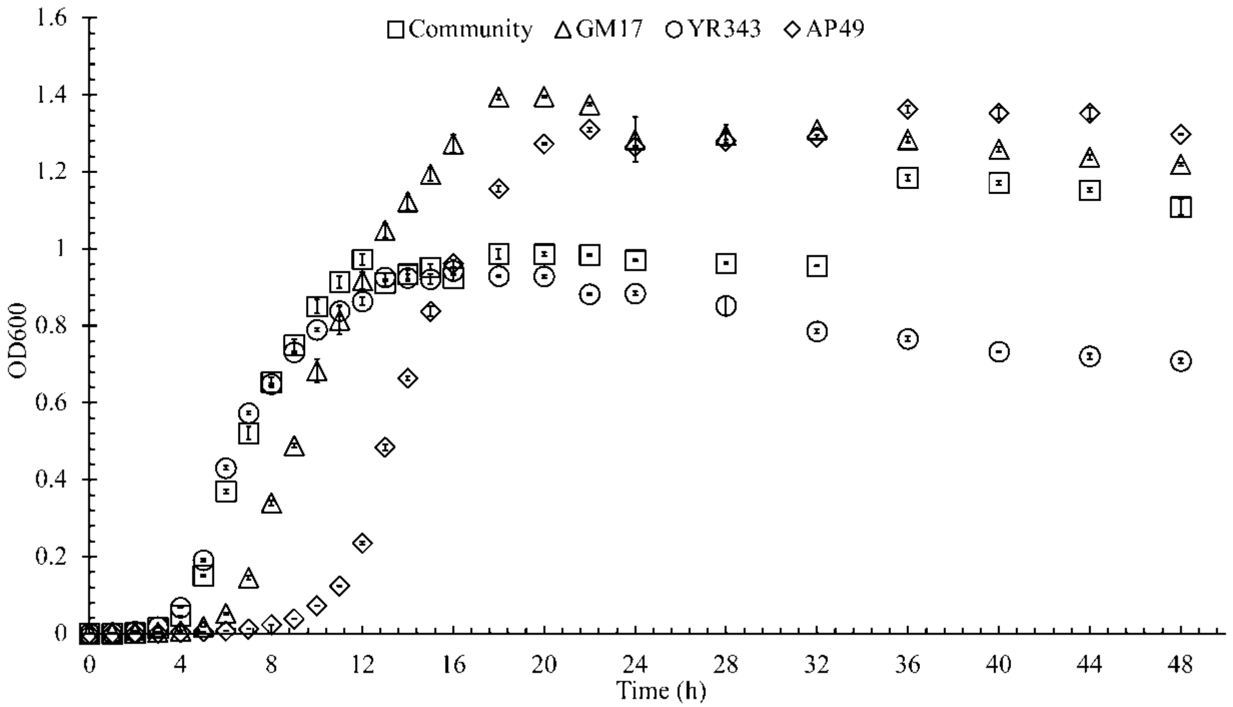

Figure S1. Growth profile of each species as individual culture and the overall growth in Passage #0 in tri-culture community starting from equal ratio analyzed by optical density. Each data point represents the mean and error bars are the standard error over three parallel experiments.

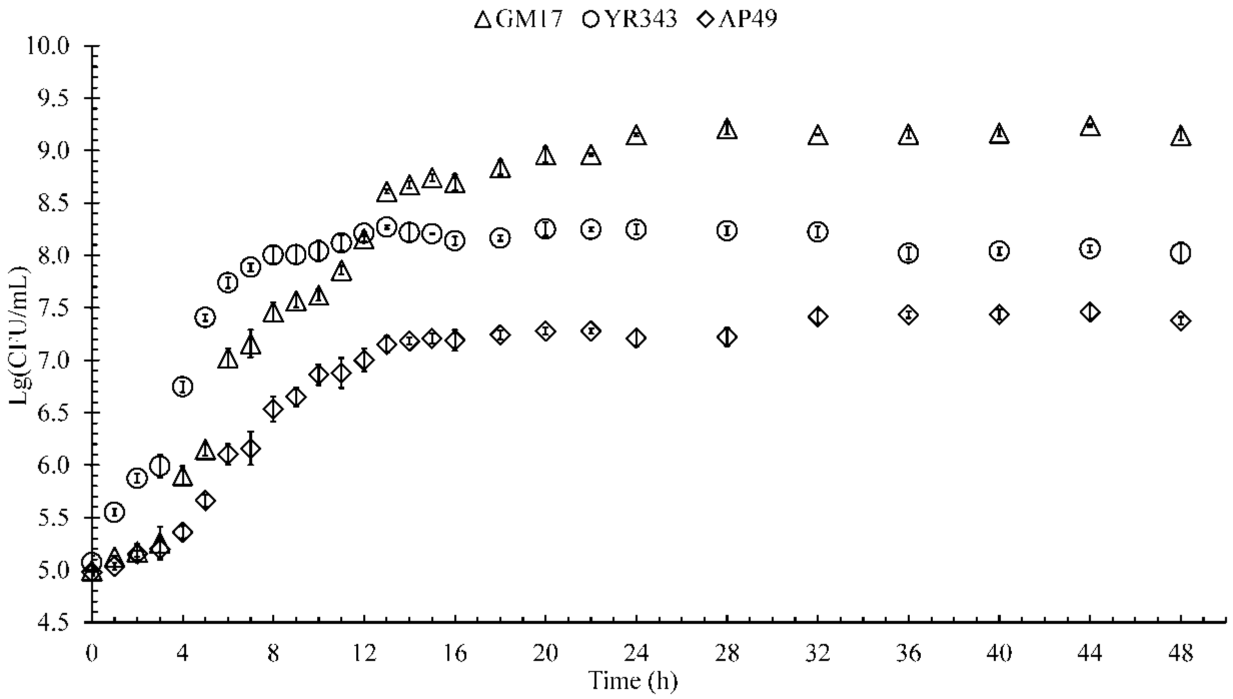

Figure S2. Growth profile of each member in Passage #0 in tri-culture community starting from equal ratio analyzed by plate counting approach. Each data point represents the mean and error bars are the standard error over three parallel experiments.

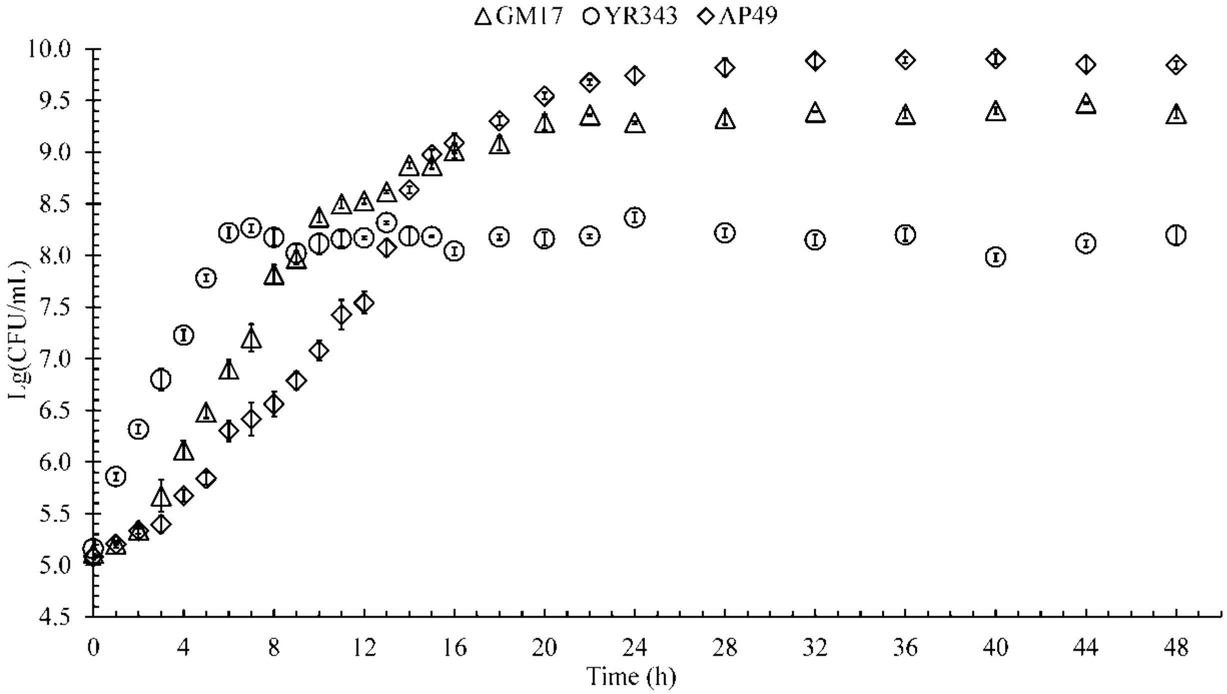

Figure S3. Growth profile of each species as individual culture analyzed by plate counting approach. Each data point represents the mean and error bars are the standard error over three parallel experiments.

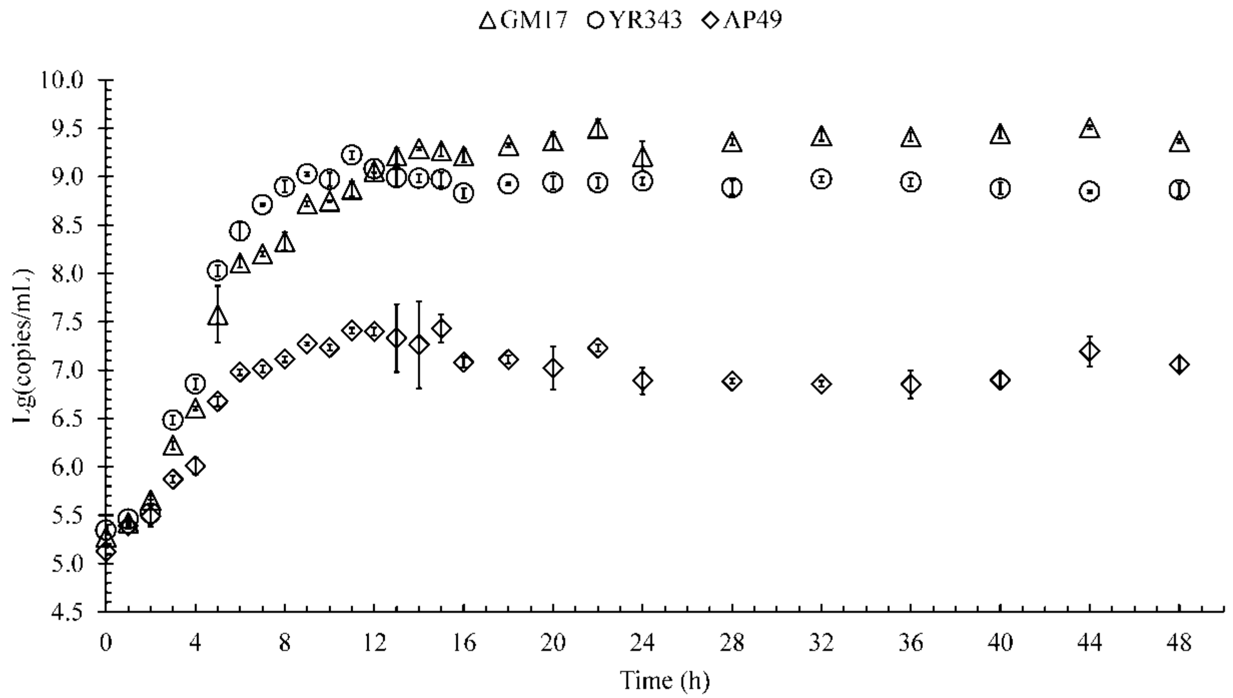

Figure S4. Growth profile of each member in Passage #0 in tri-culture community starting from equal ratio analyzed by qPCR approach. Each data point represents the mean and error bars are the standard error over three parallel experiments.

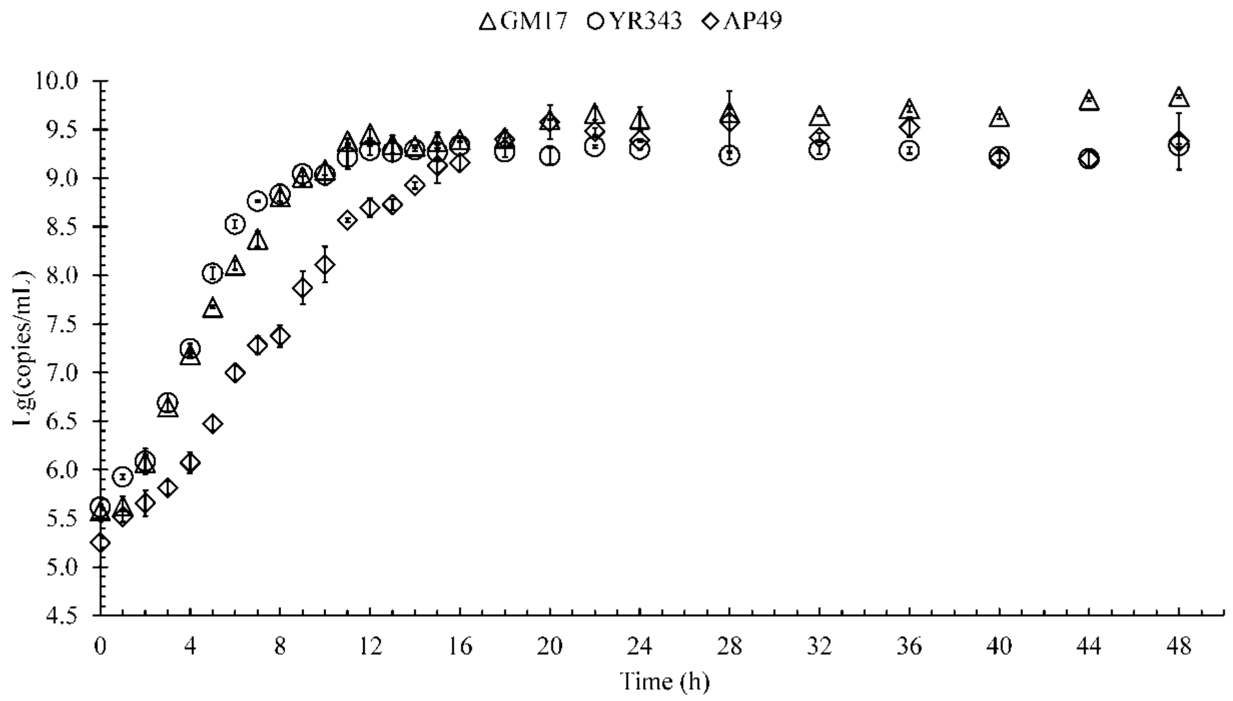

Figure S5. Growth profile of each species as individual culture analyzed by qPCR approach. Each data point represents the mean and error bars are the standard error over three parallel experiments.

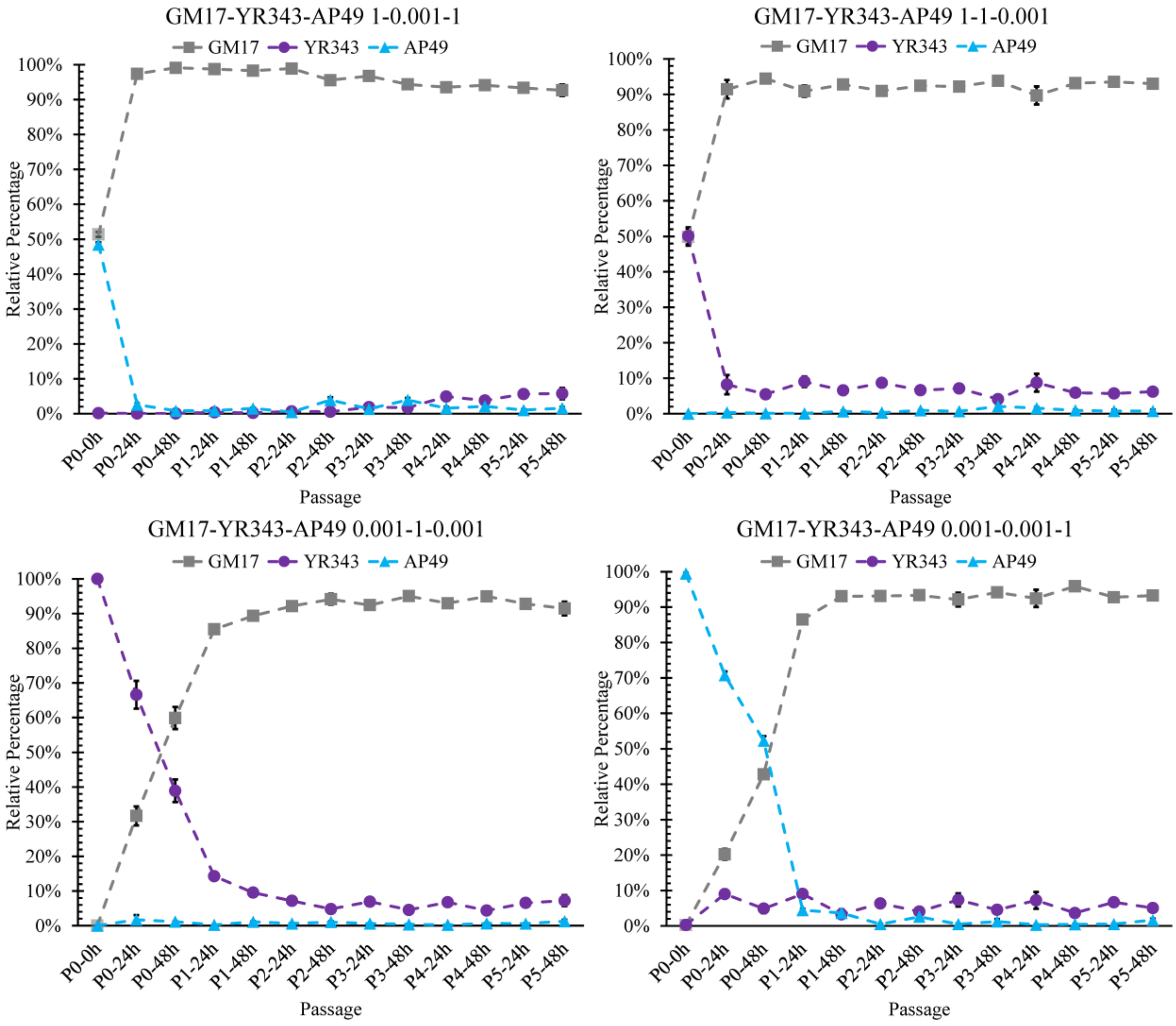

Figure S6. Snapshots of the relative percentages of each species in tri-culture communities with different initial inoculum ratios as a function of time analyzed by plate counting. Each data point represents the mean and error bars are the standard error over three parallel experiments.

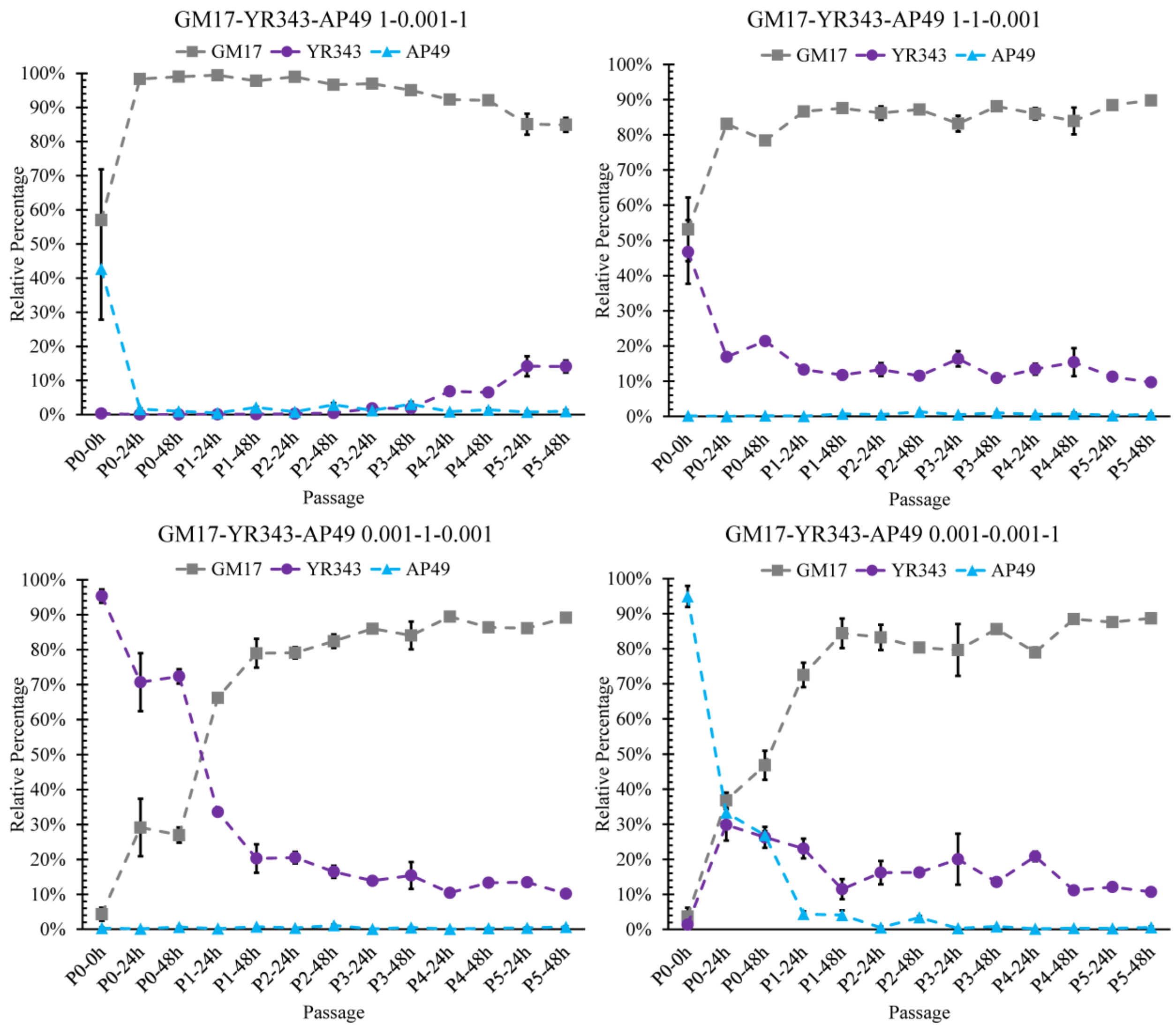

Figure S7. Snapshots of the relative percentages of each species in tri-culture communities with different initial inoculum ratios as a function of time analyzed by qPCR. Each data point represents the mean and error bars are the standard error over three parallel experiments.

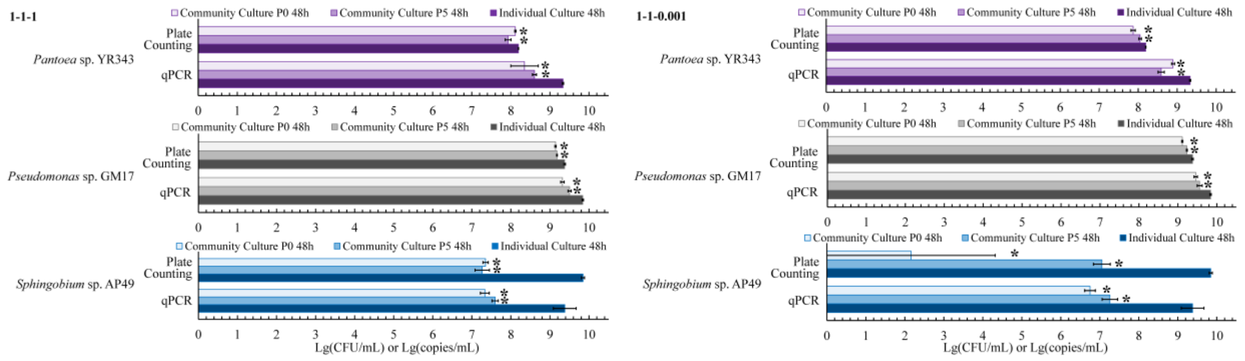

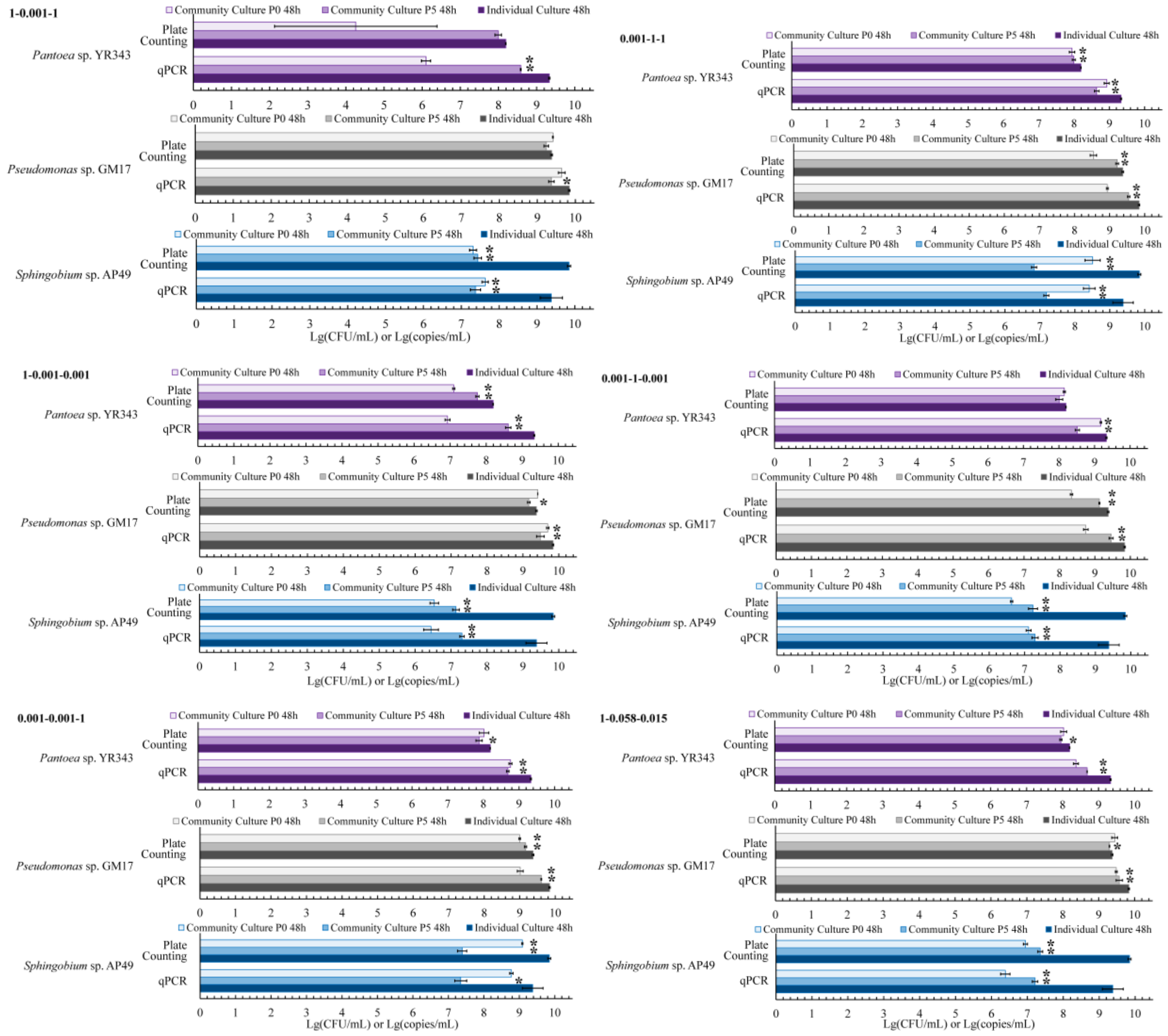

Figure S8. Comparison of the bacterial cell density values at the end of Passage #0 and Passage #5 in tri-culture communities starting from eight different initial inoculum ratios with the cell density of individual culture at 48h. Asterisks show statistically significant decrease in cell abundance compared with the corresponding monoculture using the same analytical method ($p < 0.05$). Each column represents the mean and error bars are the standard error over three parallel experiments.

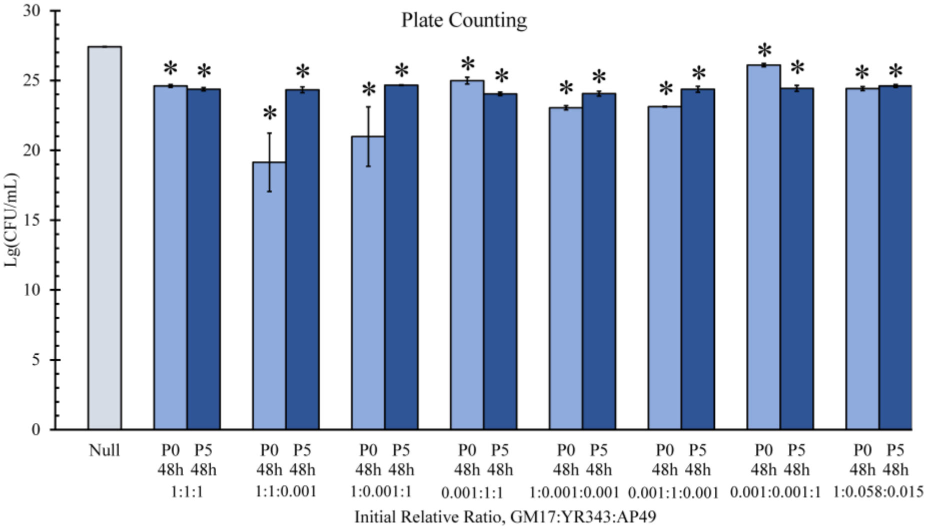

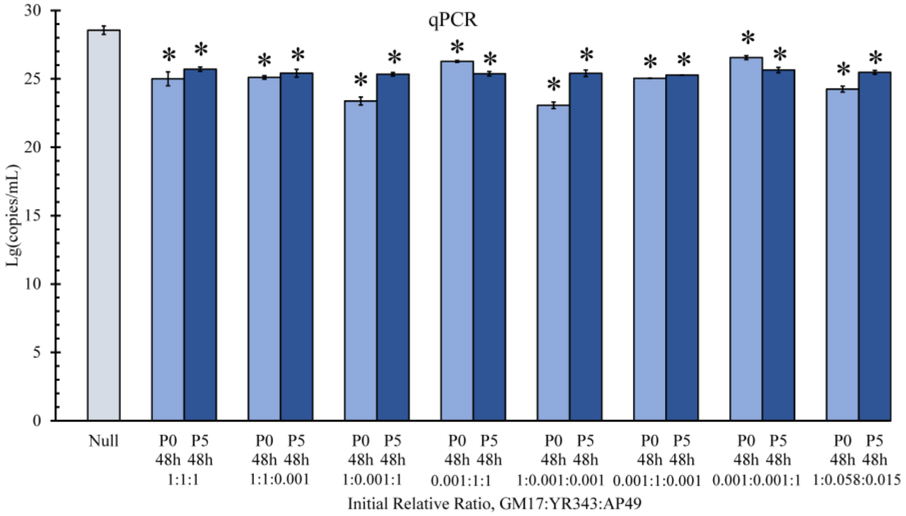

Figure S9. Comparison of the cell productivity at the end of Passage #0 and #5 in 3-member communities starting from eight different initial inoculum ratios with the cell productivity predicted by null model. Asterisks indicate statistically significant decrease in cell productivity compared to the null model prediction using the same analytical method ($p < 0.05$). Each column represents the mean and error bars are the standard error over three parallel experiments.

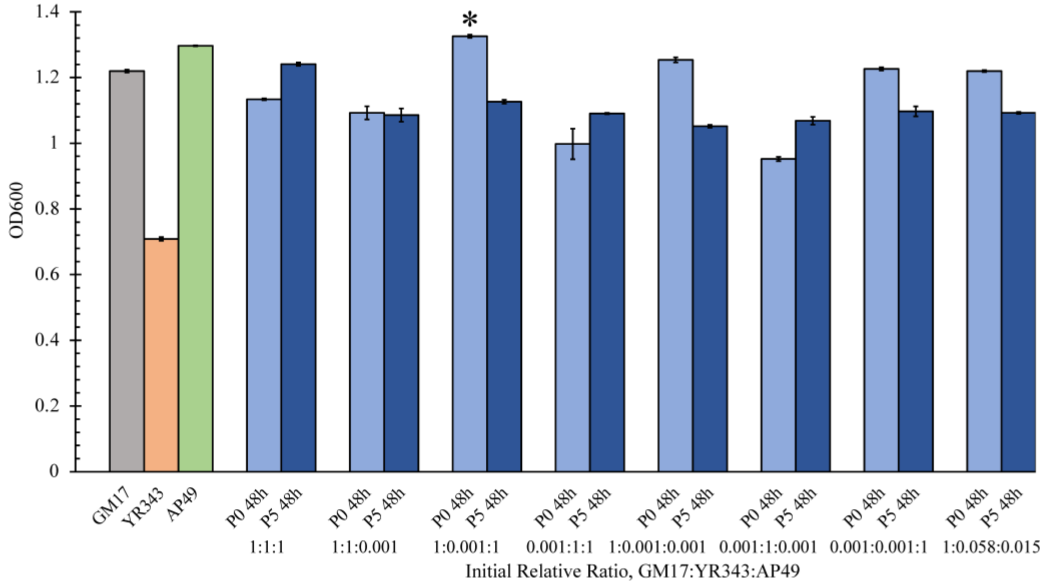

Figure S10. Comparison of the overall biomass at the end of Passage #0 and #5 in 3-member communities starting from eight different initial inoculum ratios with the growth level of monocultures. Asterisk indicates a statistically higher growth level compared to the monoculture of *Sphingobium* sp. AP49 ($p < 0.05$). Each column represents the mean and error bars are the standard error over three parallel experiments.

Calculated By Plate Counting Results

GM17 : YR343 : AP49	1:1:1	1:1:0.001	1:0.001:1	0.001:1:1	1:0.001:0.001	0.001:1:0.001	0.001:0.001:1	1:0.058:0.015
Pseudomonas to *Pantoea*	-3.0539E-10	-1.9470E-10	-2.3826E-10	-1.2208E-10	-3.0306E-10	-7.6557E-11	-3.9457E-10	-9.0530E-11
Pseudomonas to *Sphingobium*	-3.3254E-10	-1.7983E-09	-1.5783E-10	-4.4166E-10	-2.0189E-10	-3.4924E-10	-4.6319E-10	-1.2313E-10
Pantoea to *Pseudomonas*	2.3807E-10	-9.4125E-10	-1.6400E-09	-1.9089E-09	-3.5646E-09	-1.9980E-08	-1.8050E-09	-2.9050E-09
Pantoea to *Sphingobium*	1.5325E-09	-5.5848E-09	-6.9914E-10	1.6739E-09	-3.8047E-09	-1.8622E-08	9.6568E-10	-1.4069E-09
Spingobium to *Pseudonomas*	-8.6381E-09	1.6625E-09	-2.7851E-10	-1.4265E-09	-5.6344E-10	-7.5230E-08	-4.4943E-10	-2.3749E-08
Spingobium to *Pantoea*	-5.4354E-09	5.8568E-10	8.8157E-10	-4.9853E-10	-2.4542E-10	-7.9601E-09	-3.5444E-10	-2.1694E-08

Calculated By qPCR Results

GM17 : YR343 : AP49	1:1:1	1:1:0.001	1:0.001:1	0.001:1:1	1:0.001:0.001	0.001:1:0.001	0.001:0.001:1	1:0.058:0.015
Pseudomonas to *Pantoea*	-4.8897E-11	-1.1959E-10	-9.4772E-11	-1.0492E-10	-7.6620E-11	-1.2873E-10	-1.2696E-10	-2.9172E-12
Pseudomonas to *Sphingobium*	-3.8872E-11	3.4234E-11	-6.5667E-11	-1.2506E-10	-7.3115E-11	-6.0485E-11	-1.9253E-10	-1.2355E-11
Pantoea to *Pseudomonas*	-8.0853E-10	-3.8640E-10	-7.7641E-10	-3.4064E-10	-6.9172E-10	-3.8278E-10	-1.3451E-09	-9.8125E-10
Pantoea to *Sphingobium*	-1.9743E-10	-6.7057E-10	-5.1724E-10	-1.3282E-10	-7.1186E-10	-1.9385E-10	-4.5806E-10	-5.2409E-11
Spingobium to *Pseudonomas*	1.1463E-09	2.1378E-09	-2.1562E-09	-5.5479E-10	1.1389E-09	5.0373E-09	3.7643E-10	1.5371E-09
Spingobium to *Pantoea*	2.1753E-09	2.0168E-09	-3.4777E-11	-2.8130E-10	2.0062E-10	2.8875E-09	5.5680E-10	1.4672E-09

Figure S11. Heatmap of interaction coefficient (a_{ij}) values across all tri-culture communities with different initial inoculum ratios predicted by gLV model (red, negative interaction; green, positive interaction). The unitless interaction coefficient quantifies the strength of interaction between two species (j to i) in the community.

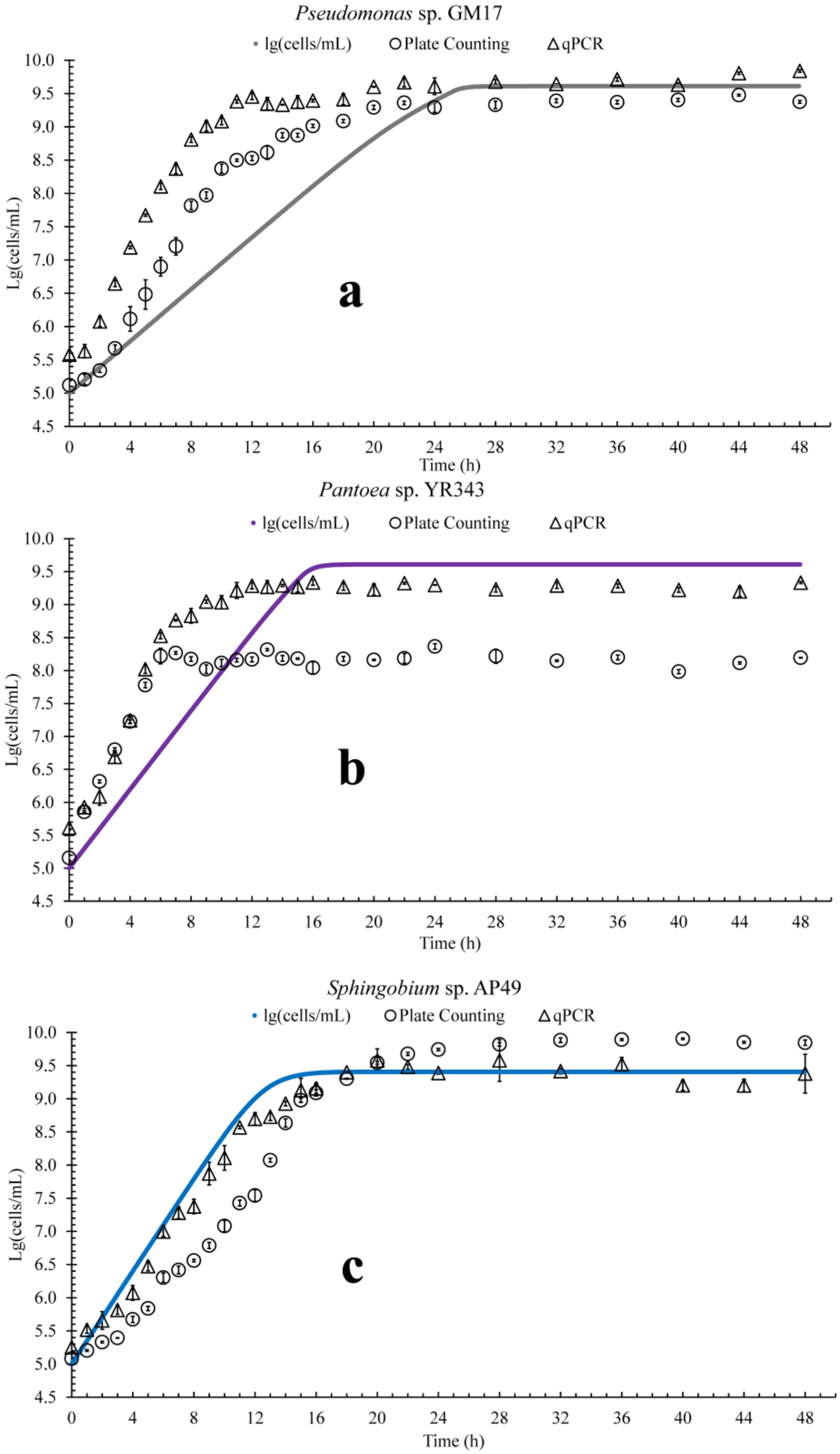

Figure S12. Validation of dFBA via comparison of growth of each species (a) *Pseudomonas* sp. GM17, (b) *Pantoea* sp. YR343, and (c) *Sphingobium* sp. AP49 predicted by dFBA and

experimental results in the pure culture growth mode. Continuous curves are time course of natural log of cell densities predicted by dFBA model of each strain in pure culture mode grown in R2A medium environment. Points are experimental data obtained from monoculture growth experiments. Each data point represents the mean and error bars are the standard error over three parallel experiments.

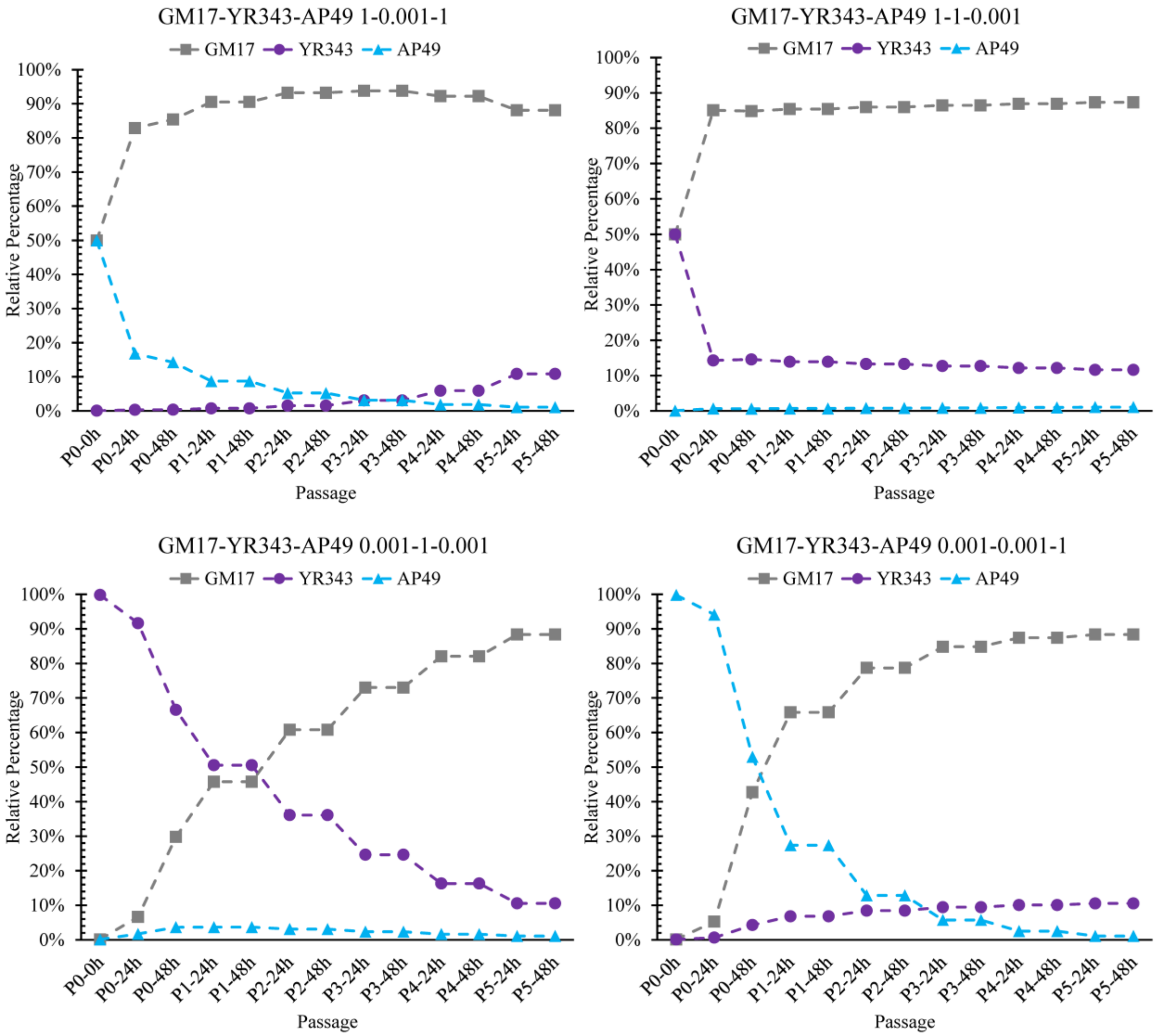

Figure S13. Snapshots of the relative percentages of each species in tri-culture communities with different initial inoculum ratios as a function of time simulated by dynamic flux balance analysis (dFBA).

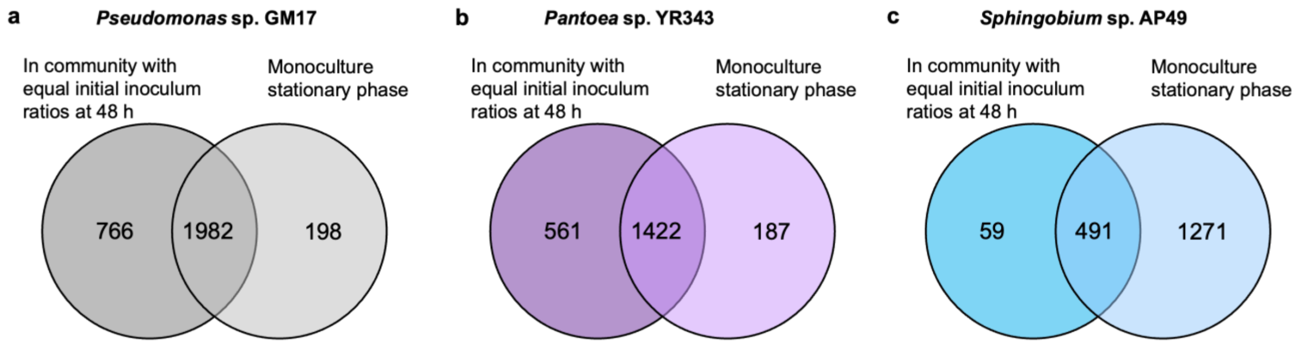

Figure S14. Venn diagrams showing the number of quantifiable proteins that are uniquely identified or shared between the three bacterial strains in the three-member community with equal initial inoculum ratios (GM17-YR343-AP49 1-1-1) at 48 h and their monocultures at early stationary phase in R2A medium (a) *Pseudomonas* sp. GM17 (b) *Pantoea* sp. YR343 (c) *Sphingobium* sp. AP49.

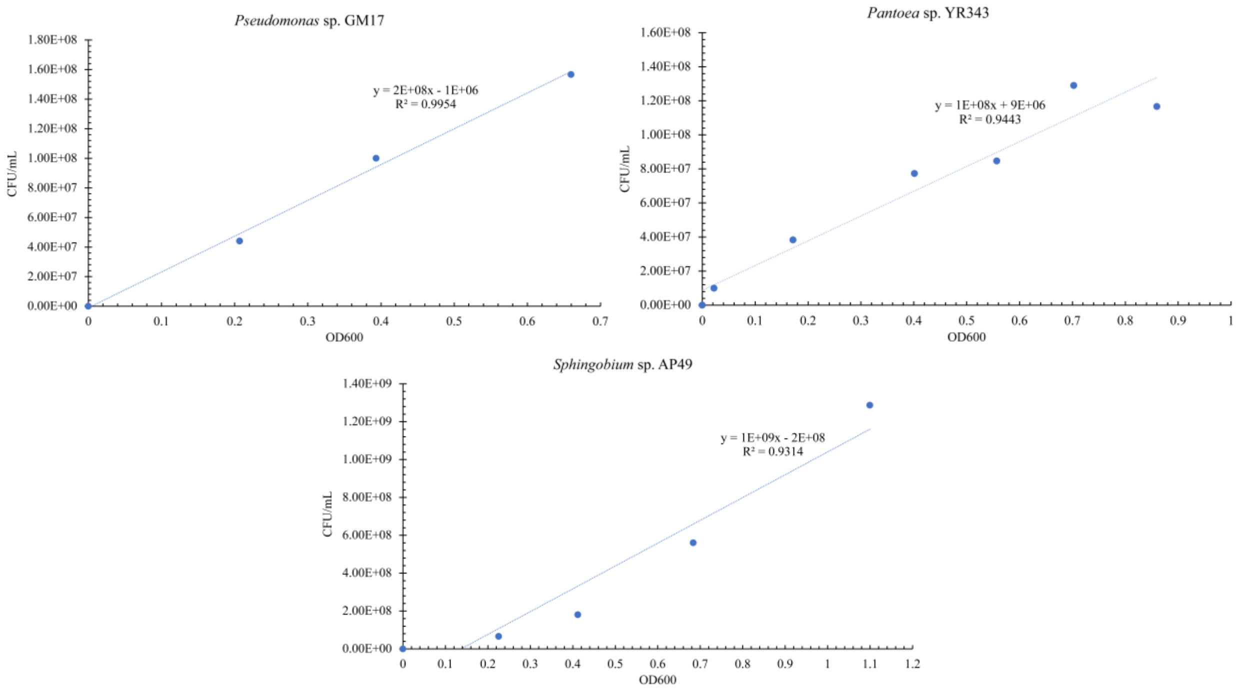

Figure S15. Calibration curve between OD600 and CFU/mL for each individual strain.

References

1. Henning JA, Weston DJ, Pelletier DA, Timm CM, Jawdy SS, Classen AT. 2019. Relatively rare root endophytic bacteria drive plant resource allocation patterns and tissue nutrient concentration in unpredictable ways. American Journal of Botany 106:1423-1434.
